# Graphene Oxide Significantly Modifies Cardiac Parameters and Coronary Endothelial Reactivity in Healthy and Hypertensive Rat Hearts *Ex Vivo*

**DOI:** 10.1021/acsomega.4c02291

**Published:** 2024-06-18

**Authors:** Marcin Z. Krasoń, Anna Paradowska, Sławomir Boncel, Mateusz Lejawa, Martyna Fronczek, Joanna Śliwka, Jerzy Nożyński, Piotr Bogus, Tomasz Hrapkowicz, Krzysztof Czamara, Agnieszka Kaczor, Marek W. Radomski

**Affiliations:** † Silesian Park of Medical Technology Kardio-Med Silesia, Marii Skłodowskiej-Curie 10C, 41-800 Zabrze, Poland; ‡ Department of Cardiac, Vascular and Endovascular Surgery and Transplantology, Silesian Center for Heart Disease, Medical University of Silesia in Katowice, Marii Skłodowskiej-Curie 9, 41-800 Zabrze, Poland; § Department of Organic Chemistry, Bioorganic Chemistry and Biotechnology, Faculty of Chemistry, Silesian University of Technology, Krzywoustego 4, 44-100 Gliwice, Poland; ∥ Centre for Organic and Nanohybrid Electronics (CONE), Silesian University of Technology, Konarskiego 22B, 44-100 Gliwice, Poland; ⊥ Department of Pharmacology, Faculty of Medical Sciences in Zabrze, Medical University of Silesia in Katowice, Jordana 38, 41-808 Zabrze, Poland; # Jagiellonian Centre of Experimental Therapeutics (JCET), Jagiellonian University, M. Bobrzyńskiego 14, 30-348 Kraków, Poland; ∇ Faculty of Chemistry, Jagiellonian University, Gronostajowa 2, 30-387 Kraków, Poland; ○ Department of Anatomy, Physiology and Pharmacology, College of Medicine, University of Saskatchewan, 107 Wiggins Rd, Saskatoon SKS7N 5E5, Canada

## Abstract

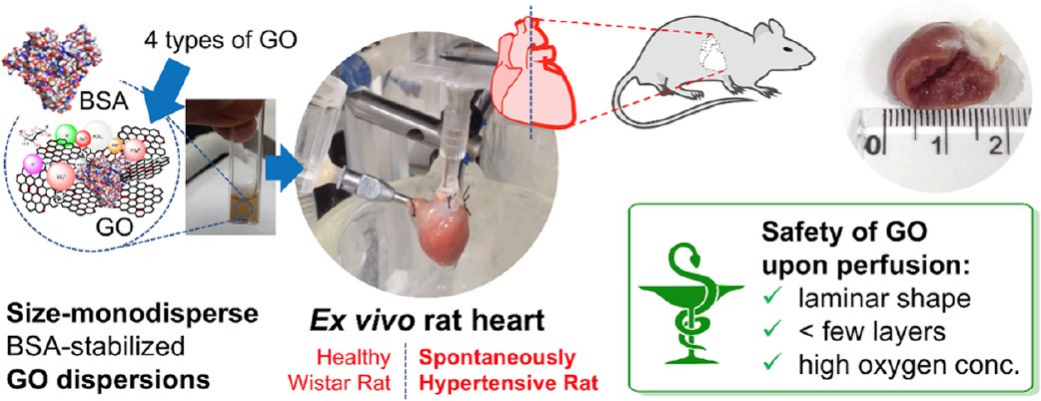

Interactions of graphene oxide (GO) with an *ex vivo* rat heart and its coronary vessels have not been studied yet. Moreover, the conflicting data on the “structure-properties” relationships do not allow for biomedical applications of GO. Herein, we study the impact of GO on the *ex vivo* isolated rat heart, normotensive and hypertensive, under the working heart and the constant-pressure perfusion (Langendorff) regimes. Four structural GO variants of the following initial morphology were used: few-layer (below 10-layer) GO1, O < 49%; predominantly single-layer GO2, O = 41–50%; 15–20-layer GO3, O < 11%; and few-layer (below 10-layer) NH_4_^+^-functionalized GO4, O < 44%, N = 3–6%. The aqueous GO dispersions, sonicated and stabilized with bovine serum albumin in Krebs–Henseleit-like solution—uniformized in terms of the particle size—were eventually size-monodisperse as revealed by dynamic light scattering. To study the cardiotoxicity mechanisms of GO, histopathology, Raman spectroscopy, analysis of cardiac parameters (coronary and aortic flows, heart rate, aortic pressure), and nitric oxide (NO−)-dependent coronary flow response to bradykinin (blood-vessel-vasodilator) were used. GO1 (10 mg/L) exerted no effects on cardiac function and preserved an increase in coronary flow in response to bradykinin. GO2 (10 mg/L) reduced coronary flow, aortic pressure in normotensive hearts, and coronary flow in hypertensive hearts, and intensified the response to bradykinin in normal hearts. GO3 (10 mg/L) reduced all parameters in hypertensive hearts and coronary response to bradykinin in normal hearts. At higher concentrations (normotensive hearts, 30 mg/L), the coronary response to bradykinin was blocked. GO4 (10 mg/L) reduced the coronary flow in normal hearts, while for hypertensive hearts, all parameters, except the coronary flow, were reduced and the coronary response to bradykinin was blocked. The results showed that a low number of GO layers and high O-content were safer for normal and hypertensive rat hearts. Hypertensive hearts deteriorated easier upon perfusion with low-O-content GOs. Our findings support the necessity of strict control over the GO structure during organ perfusion and indicate the urgent need for personalized medicine in biomedical applications of GO.

## Introduction

Graphene oxide (GO), in its numerous morphological and physicochemical variants, is an important nanomaterial for biomedical applications, while also being able to affect the cardiovascular system. Biocompatibility of GO with diverse cells, tissues, and organs depends on the oxygen content,^1^ particle size and shape,^2^ and the number of layers.^3^ Tunable, covalent, and noncovalent functionalization of GO can lead to more promising drug transport capabilities by reducing the toxicity of GO and improving its water dispersibility, which is further hampered at high ionic strength.^4^ The key promising biological properties of GO include antibacterial^5^ activity that can be effective also against multidrug-resistant bacteria,^6,7^ particle hydrodynamics and physicochemistry toward drug delivery platforms,^8^ including the oral drug administration route,^9^ and structural characteristics enabling scaffolding tissues in regenerative medicine.^10^ Recently, GO-structured gels have been tested to reinforce mechanically myocardial postinfarct scars,^11^ ammonia-functionalized graphene/GO or the smallest graphene quantum dots are being tested in regenerative medicine,^12^ and further in the antibacterial therapies.^13^ Those multiple prospective applications lead inevitably, in the forthcoming future, to a potentially increased presence of GO in the human cardiovascular system and tissues.

The toxicity of GO was evaluated *in vitro* in cellular models for adherent^14^ and nonadherent^15^ cell types and was reported to be low or moderate. Furthermore, GO (only particles of a small lateral size) increased the growth rate of the vascular smooth muscle cells while not affecting cell migration.^16^ Other reports described oral applications of GO that displayed potentially positive effects on the intestines with no adverse impact on other organs^9^ or, contrarily, time-dependent proinflammatory effects on the lungs after several days of inhalation.^17^ The damage to the critical organs and the GO distribution after intravenous administration were evaluated in animal studies,^18−20^ but the results should be analyzed with caution since GO dispersions were prepared in water^18,19^ or saline^20^ and did not contain Ca^2+^ or Mg^2+^. Such an approach could cause unknown changes in the GO morphology, including a Ca^2+^-induced aggregation and modification of the number of GO layers at the moment of direct contact of GO with organs or tissues. Presumably, the troublesome dispersion stability of GO in the ionic solutions is one of the most important obstacles in testing its influence on the circulatory and cardiac parameters. The instability of the dispersion can possibly modify the particle effects by lowering the effective blood concentration by aggregation at the injection site^20^ or at the first line of organ capillaries that can effectively filter the largest particles.^19^ This hindrance derives from the fact that the blood and isotonic perfusion buffers—used in the *ex vivo* heart perfusion studies—contain relatively high concentrations of calcium, magnesium, and sodium cations^21−25^ that can instantly precipitate GO *in vitro* and shortly after a tissue or vein injection.^19,20^ The aggregation of GO was observed at similar to isotonic levels of bivalent calcium and/or magnesium^26,27^ or monovalent sodium cations,^28^ despite a high dispersibility of GO in water and the initial stability of such dispersions. The colloid stability of GO is improved with the increasing number of oxygen-containing functional groups; it can be modified by other functionalization routes or by changing their pH-dependent ionization degree.^29,30^

At the same time, microvessels free from aggregates, for instance, those precipitated from colloids, are vital for effective blood circulation and the preservation of organ function. It is especially important in the heart because cardiac tissue is highly dependent on coronary flow,^31^ vessel structure, and endothelial function.^32^ The coronary flow is regulated by the constant interplay of endothelium-produced vasoconstrictors and vasodilators. The major endothelium-derived relaxing factors are nitric oxide (NO), prostaglandins, and hyperpolarizing factors. The latter ones are heterogeneous in nature^33^ and include hydrogen sulfide (H_2_S), which is important in endothelial dysfunction in diabetes.^34^ The function of the endothelium, which is crucial in the pathogenesis of hypertension,^35−37^ and NO–GO interaction at the intact coronary vascular level, has not been evaluated yet, in either normotensive or hypertensive hearts. The endothelial function is becoming increasingly important also in ischemic heart disease with diagnosed stenosis of coronary vessels, newly reported nonobstructive coronary artery ischemic disorders, heart failure with preserved ejection fraction, and other noncardiac conditions, such as chronic inflammatory disorders or liver diseases.^38^ The coronary flow reserve test (with acetylcholine as a vasodilator) is a recommended tool for invasive assessment of nonobstructive coronary artery disease^39^ with microcirculatory, NO-dependent disorders. A diminished response to acetylcholine in vascular rings, reported in spontaneously hypertensive rats (SHR) *ex vivo*, suggested lower activity of endothelium-derived relaxing factor (EDRF) or NO as compared to the normotensive Wistars.^40^ The possible mechanisms of a gradual induction of hypertension in SHRs are multifactorial and not fully understood, although, they resemble human hypertension. Some of them are related to free radicals, metabolic deficiencies (a level of tetrahydrobiopterin, *i.e.*, a cofactor important in the NO synthesis, which is reduced in male SHRs),^41^ or neurogenic factors.^42^ Rarefaction of microvessels^43^ found in SHRs was also observed in hypertensive humans^44^ as well as the left ventricle hypertrophy which accompanies the hypertension development in animals. Since some toxic effects of GO depend on the harmful mechanical interaction with cells that can be related to defined particle features,^45^ and/or generation of free radicals,^46^*i.e.*, both reactive oxygen (ROS) and nitrogen species (RNS), as well as the concentration-dependent activation of free radicals scavenging enzymatic systems,^18^ bradykinin-induced NO-dependent coronary vasodilation might be modified in the presence of GO. Due to a lack of data evaluating the influence of GO on the NO-dependent coronary vasodilation, bradykinin^47^ and indomethacin^48^ (a blocker of the synthesis of prostaglandins, non-NO-dependent vasodilators) could be used to study NO-dependent endothelial vasodilation in the presence of GO instead of the acetylcholine, which when injected into the animal bloodstream can induce both tachycardias and bradycardias as well as the arterial pressure increase.^49^

Here, toward a better understanding of the nature of the effective interactions between GO and coronary vasculature of contracting heart, four types of GO particles, including a GO type containing a portion of positively charged ammonium groups (NH_4_^+^), were studied as aqueous dispersions containing high concentrations of calcium and magnesium cations, (*i.e.*, isotonic Krebs–Henseleit (KH) buffer), in an *ex vivo* perfusion model of isolated rat heart. The stability of GO dispersions was accomplished using the original protocol of sonication and dilution with the pretested and optimized concentrations of bovine serum albumin (BSA).^50^ The GO dispersions were further applied to investigate their qualitative and quantitative impact on the normotensive (Wistar) and hypertensive (SHR) *ex vivo* rat hearts. The studies were performed in the regime of working heart and the constant-pressure perfusion (Langendorff) revealing the least and most cardio-toxic types of GO, in terms of morphology and surface physicochemistry.

## Experimental Section

### Materials

Four different commercially available aqueous GO dispersions were used as masterbatches without further purification: (1) few-layer (<10 layers) GO1 (Nano Carbon LLC, Warsaw, Poland), 5 mg mL^–1^, C:>45%, H:<2.5%, N:<0.5%, O:<49%, S:2.5%, others:<1.5%; (2) single-layer (95%) GO2 (Graphenea, San Sebastian, Spain; https://www.graphenea.com), 4 mg mL^–1^, C:49–56%, H:0–1%, N:0–1%, O:41–50%, S:2–4%; (3) 15–20-layer GO3 (Garmor, Inc., Orlando; purchased from Sigma-Aldrich, Poznań, Poland), 1 mg mL^–1^, C: ≥50%, O: ≤11%; and (4) few-layer (<10 layers) NH_4_^+^-functionalized GO4 (Sigma-Aldrich, Poznań, Poland), 1 mg mL^–1^, C:40–50%, N:3–6%, S: ≤3%. BSA (99.9% purity) was purchased from BLIRT, Gdańsk, Poland (https://blirt.pl; Qiagen Gdańsk sp. z o.o.). Ultrapure water (Lichrosolv liquid chromatography–mass spectrometry (LC–MS) water, Merck, Darmstadt, Germany) was purchased from Merck, Poznań, Poland. The reagents for the modified isotonic Krebs–Henseleit (KH) solution (NaCl, KCl, NaHCO_3_, KH_2_PO_4_, anhydrous CaCl_2_, anhydrous glucose–all analytical grade) were purchased from Avantor Performance Materials, Gliwice, Poland. Analytical grade anhydrous MgSO_4_ was purchased from Sigma-Aldrich, Warsaw, Poland. Sodium pyruvate (99%+) was purchased from Acros Organics, Belgium, now: Thermo Scientific Chemicals, Hague, The Netherlands. Bradykinin chloride and indomethacin crystalline were purchased from Sigma-Aldrich, Poznań, Poland.

### Experimental Animals

The experiments were conducted in compliance with the EU Directive 2010/63/EU for animal experiments and according to the protocol approved by Local Bioethics Committee for Animal Use at the Medical University of Silesia (decision no. 75/2016 of 14th June 2016) on 90 Wistar adult male rats (mean weight 450 ± 3 g) and 40 Spontaneously Hypertensive Rats (SHR) adult males (mean weight 310 ± 1 g). The animals were supplied from Charles River Laboratories (Freiburg, Germany) and kept for 4 weeks (Wistar rats) and at least 6 weeks (SHRs) under standard conditions (dark/light 12/12 h; 21–23 °C in double cages, with an unlimited access to the standard chow and water). SHRs have been checked for hypertension. The measurement of arterial blood pressure was performed after the adaptation to the measuring tube twice a week using the Rat Blood Pressure Monitor (IITC Life Science; Woodland Hills, CA) with the dedicated software. SHRs were enrolled in the study when their systolic blood pressure was >140 mmHg. Wistar rats were screened for hypertension and excluded from the study cohort when their systolic blood pressure reached >140 mmHg.

### Experimental Protocol

#### Preparation of GO Dispersions, Dilutions, and Mixtures

To prepare GO samples, stock GO dispersions were shaken several times, and the volumes of 1.4 mL (GO1), 1.75 mL (GO2), 7 mL (GO3), and 7 mL (GO4) were transferred to 75 mL beakers, and dispersed to the final volume of up to 50 mL with ultrapure water (GO at a concentration of 140 μg mL^–1^). Then, it was sonicated, mixed with separately prepared BSA suspension, and then with solution containing the aggregating cations to achieve the final solution containing 10 μg mL^–1^ GO (GO1, GO2, GO3, and GO4) or 30 μg mL^–1^ (GO2 and GO3); BSA 80 or 240 mg mL^–1^, respectively; NaCl (124 mmol L^–1^), KCl (4.2 mmol L^–1^), NaHCO_3_ (15 mmol L^–1^), MgSO_4_ anhydrous (1.5 mmol L^–1^), KH_2_PO_4_ (1.2 mmol L^–1^), CaCl_2_ anhydrous (1.5 mmol L^–1^), sodium pyruvate (5.0 mmol L^–1^), and anhydrous glucose (5.6 mmol L^–1^). A microvolume of bradykinin stock solution was added to 200 mL of the solution (the final bradykinin concentration was 100 nM, while indomethacin 5 μM). For details, see the Supporting Information (SI).

#### Heart Preparation and Perfusion, Data Collection

**Figure 1:**
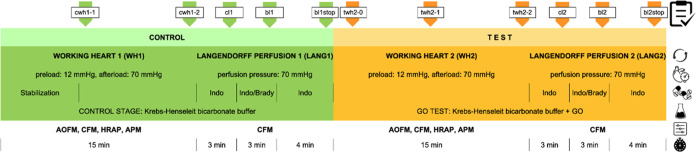
Perfusion protocols. Abbreviations: cwh1–1, cwh1–2—working heart control parameter recording periods (40 s); cl1, bl1, bl1stop—Langendorff perfusion control parameter recording periods (40 s); twh2–0, twh2–1, twh2–2—working heart GO study parameter recording periods (40 s); cl2, bl2, bl2stop—Langendorff perfusion GO study parameter recording periods. cl, bl, blstop stand for respectively: control Langendorff period, bradykinin administration period, post-bradykinin administration period. Indo, indomethacin (5 μM) perfusion, Indo brady: perfusion with indomethacin (5 μM) and bradykinin (100 nM); AoFM, aortic flow mean; CFM, coronary flow mean; HRAP, HR, heart rate from aortic pressure trace; APM, aortic pressure mean.

Animals were administered heparin intraperitoneally (5000 IU) 60 min before anesthesia, and later they were anesthetized intraperitoneally with xylazine (10 mg kg^–1^) and ketamine (100 mg kg^–1^). When the animals stopped moving, they were transferred to the operating table and the degree of anesthesia was monitored with delicate touching the paws with pincette. When the animals stopped reacting to any stimuli, the chest was opened, and the heart was excised together with the lungs. The harvested tissues were cooled in the ice-cold KH buffer. After a short preparation, the aorta was cannulated, and perfusion of the heart was restarted. The lungs were removed, and the pulmonary artery and the left atrium were cannulated. After the left atrial cannulation, the perfusion pressure was lowered to 12 mmHg, and the working heart mode of perfusion was initiated (Figure 1).

To reduce the number of animals in the study, each heart served as its own control. All hearts were perfused both with standard Krebs–Henseleit buffer (Figure 1, control stage in green) and in the second part of the protocol with the same buffer enriched with the tested GO (Figure 1, test stage in orange). The parameters of the working heart control stage (Figure 1, in green: cwh1–2) were compared with the corresponding parameters in the test stage (Figure 1, in orange: twh2–0, twh2–1, twh2–2) in the same group. Both in control (green) and test (orange) protocol stages, two perfusion regimes (*i.e.*, Langendorff-constant pressure and working heart) were used. To assess the bradykinin vasodilating effect on coronary vessels objectively, the bradykinin test was performed twice (with and without GO). The mean coronary flow during bradykinin infusion (bl stages) was compared to pre-bradykinin control perfusion period (cl stages) separately in green and orange experiment periods (Figure 1). In “bl” and “cl” stages of the experiments, control parameters were collected in the third minute both in the “LANG1” control stage—“cl1” and in the “LANG2” test stage—“cl2”, and were compared, respectively, with “bl1” and “bl1stop” (“LANG1”) and “bl2”, “bl2stop” (“LANG2”). Additionally, parameters “bl” and “blstop” in control and test stages were expressed as percent of their control values (see results), and only these values were compared between the studied groups, *i.e.*, GOs. The control and working heart test stages were not compared between the groups (*i.e.*, between the different types of GO).

The hearts were accepted for GO perfusion when the mean aortic flow (AoFM) was at least 35 mL min^–1^ in Wistar rats and >25 mL min^–1^ in SHRs at the measuring point (“cwh1–1” or “cwh1–2”). The data were obtained using the PLUGSYS system (HSE, Hugstetten, Germany), digitized with Power LAB 8 (Adinstruments, Colorado Springs), and recorded every 2 s using Lab Chart Pro software (Adinstruments, Colorado Springs) at a sampling rate of 1000 s^–1^. All calibrations, perfusion dispersions/solutions, and GO sonication were prepared or performed in the morning of the experimental day. The perfusion KH buffer was filtered with 5 μm filters (Pall Medical HP1050, Warsaw, Poland) immediately before reaching the perfusion system. Carbogen used for oxygenation of the KH buffer (95% O_2_ and 5% CO_2_) was filtered with dedicated medical gas filters (Pall Medical HP2002, Warsaw, Poland). Ten study groups, with number of experiments from 6 to 8 were defined according to the GO type, rat strain, and concentration of the tested GO (GO2 and GO3 at concentrations of 10 and 30 mg mL^–1^ due to their different behavior) (Table 1).

**Table 1:** *Ex Vivo* Rat Heart Study Groups^a^

study group	no. of experiments	animal strain	GO concentration (μg mL^–1^)	BSA concentration (mg mL^–1^)
G1–10	6	wistar	10	80
G2–10	6			
G3–10	6			
G4–10	6			
G1–10SHR	6	SHR	10	80
G2–10SHR	7			
G3–10SHR	7			
G4–10SHR	7			
G2–30	7	wistar	30	240
G3–30	8			

a G1–4 represent here the name of the study group, whereas GO1–4 represent the names of the used GOs.

#### Measurements of Hydrodynamic GO Diameters in Dispersions

The hydrodynamic diameter of the GO particles in the final dispersions was measured using dynamic light scattering (DLS) (Zetasizer Nano ZS, Malvern Instruments, Malvern, England). The scattered light was measured for 1 mL samples at an angle of 173°, at 37 °C, after the stabilization time of 120 s. Upon completion of the measurement, the material was returned to the main sample. The averaged results representative for all groups were calculated by the Zetasizer software from at least 10 measurements.

#### Histopathology

To perform histological assessment, six (6) hearts were stained from the following groups: G1–10, G2–10, G3–10 (two hearts), G4–10, and G3–30, while one (1) heart was perfused with GO3 at a concentration of 10 μg mL^–1^ without applying the 5 μm KH particle filter. Immediately after perfusion, the hearts were dried with a soft tissue and fixed by immersion in a 4% buffered neutral formalin solution. The transverse sections were dehydrated with graded alcohol and xylene (a mixture of isomers), and embedded in paraffin. Tissue slices (4 μm) were cut using a Leica rotational microtome and routinely stained with hematoxylin and eosin (H&E).

#### Raman Spectroscopy

Raman spectroscopy was used to assess the presence of the GO particles in the heart tissues. Raman spectra of air-dried rat heart cross sections were acquired using a Confocal Raman Microscope (WITec alpha300, Ulm, Germany) equipped with a 532 nm laser, a UHTS 300 spectrograph, a charge-coupled device (CCD) detector (DU401A-BV-352, Andor, U.K.), and a 100× air objective (NA = 0.90, Olympus MPLAN). The images were taken from areas of 20 × 20 μm^2^ with a sampling density of 0.2 μm in the *x*/*y* directions. The spectra were collected with a 0.03 s exposure time per spectrum with a 10 mW laser power at the sample using cluster analysis (CA).

Raman spectra of the aqueous GO dispersions were measured on CaF_2_ slides, with the application of the 100× air objective (NA = 0.90, Olympus MPLAN, Japan). For each spectrum, 10 scans were collected with an integration time of 0.5 s and laser power of 5 mW at the sample using CA. All spectral data processing, including baseline correction, cosmic ray removal, and CA (*K*-means, Manhattan distance), was performed using the WITec Project Plus software. Raman distribution images were obtained by integration of the signals in the 3030–2830 and 1657–1538 cm^–1^ ranges, assigned to the organic matter and GO in the tissue, respectively. CA enabled grouping of the spectra from the acquired data sets into classes reflecting the area of accumulated GO (red class) and heart tissue (blue class).

#### Atomic Force Microscopy

The morphology of the pristine GO was characterized by atomic force microscopy (AFM). AFM images were obtained using a MultiMode with Nanoscope IIId controller, and a Veeco (USA) microscope equipped with a piezoelectric scanner of a scan range of 10 × 10 μm^2^. The imaging of samples was conducted in the tapping mode in ambient air conditions at a scan rate of 1 Hz using etched silicon probes (TESP, Bruker) of nominal spring constant 42 N m^–1^ and operating at a resonant frequency of 320 kHz. All samples were imaged at room temperature. Spin150 wafer spinner (Semiconductor Production Systems) was used under the following conditions: on the glass, at RT in air, 400 rpm, 60 min. Cover glasses were used (13 mm in diameter) as cleaned in acetone and in the plasma cleaner with oxygen (purity 6.0).

#### Measurement of Absorbance of GO Dispersions

The spectrophotometric analysis, with a Tecan Spark multimode microplate reader, was used to assess the losses of GO particles in (a) the heart and (b) the 5 μm perfusion system filter. The measurements were performed at 325 nm to avoid excessive background absorption of polystyrene microplates in the 200–300 nm range. After passing through the rat heart and the 5 μm filter (without the perfused heart), the samples of the perfusate were collected at the end of randomly selected experiments. The absorbance of the samples was compared to the baseline optical density at 325 nm wavelength (OD_325_) of the GO-BSA-KH dispersion, which was referenced as 100%.

#### Echocardiography in Rats

Echocardiography was applied to confirm adequate hypertension-related changes in the SHR group and, at the same time, to exclude rats with dilated or hypertrophied left ventricles from the Wistar group. It was performed at least once in every rat 1 day before the experiment with the ultrasound machine (VEVO 2100 Imaging System with transducer MS400 30 MHz). Before the examination, the animals were anesthetized with the inhalation of isoflurane (4%) in an isolated gastight chamber (VEVO Anesthesia System). Under full anesthesia, the animals were transferred to a heated platform equipped with an ECG monitor and the mask for anesthesia so that administration of isoflurane (1.5–2%) in the gas mixture could be continued. The chest fur was removed with a depilatory cream. The diameters of the left ventricle were measured in the three-chamber projection from the parasternal view in B-mode (brightness mode, standard two-dimensional (2D) image).

### Statistical Analysis

The data were presented as mean ± standard deviation (SD) in the tables and as mean ± standard error in the plots. Due to the low number of observations in each group (6–8, see Table 1), nonparametric tests were used. To compare the paired variables, the Wilcoxon signed rank exact test for the paired samples was used. To analyze differences between the independent measurements, the Wilcoxon rank sum exact test was used in the case of comparing the two groups. To compare more than two independent groups, the Kruskal–Wallis test was applied with the Dunn test for post hoc comparisons. *P* values lower than 0.05 were considered significant. The analysis was performed using the R-language in the R-studio environment.

## Results and Discussion

Our data describe nanoparticle “size- and structure-properties” effects of various GO types exerted on the mammalian working heart *ex vivo*. The content of oxygen, number of single GO layers, presence of additional ammonium groups, and the particle shapes were different among the studied nanoparticles. The size of BSA-stabilized GO particles was measured in the BSA-KH isotonic aqueous dispersions, and compared with the stock dispersions and between the studied groups.

### GO Particle Characteristics and GO Tissue Distribution

The results of the DLS particle measurements of GO are reported in Table S1 and Figure S1. Commonly, GO is reported to be well dispersed in water,^51^ and the main mechanism governing this phenomenon is the deprotonation of edge-located, oxygen functional groups, such as carboxylic and phenolic ones, with the subsequent repulsion of the negatively charged flakes or particles.^51,52^ Hence, in general, the higher the number of those functionalities, the more pronounced the GO water dispersibility. In turn, low pH and the presence of bivalent cations intensify the aggregation process.^26^ The cation critical coagulation concentrations can be lower than cation concentrations observed in physiology. Yang et al. suggested that very low concentrations of sodium (only above 30 mM), potassium (20 mM), magnesium (0.7 mM), and calcium (0.4 mM) destabilized aqueous GO dispersions by aggregation.^28^ In our study, in the preliminary tests, we found that the GOs we used instantly aggregated upon mixing with the KH buffer. The oxygen content in our samples varied between the GOs from ≤11% wt % (GO3) to 41–50% (GO1 and GO2), and those differences could be also a source of potential variations in their dispersion behavior.^30^ Here, the oxygen content corroborated the transparency of the aqueous GO dispersions (see further Figure 3, inset). Indeed, GO1 and GO2 dispersions were transparent and yellowish,^53^ while the GO3 and GO4 dispersions were much darker and not translucent at the initial concentrations.

**Figure 2:**
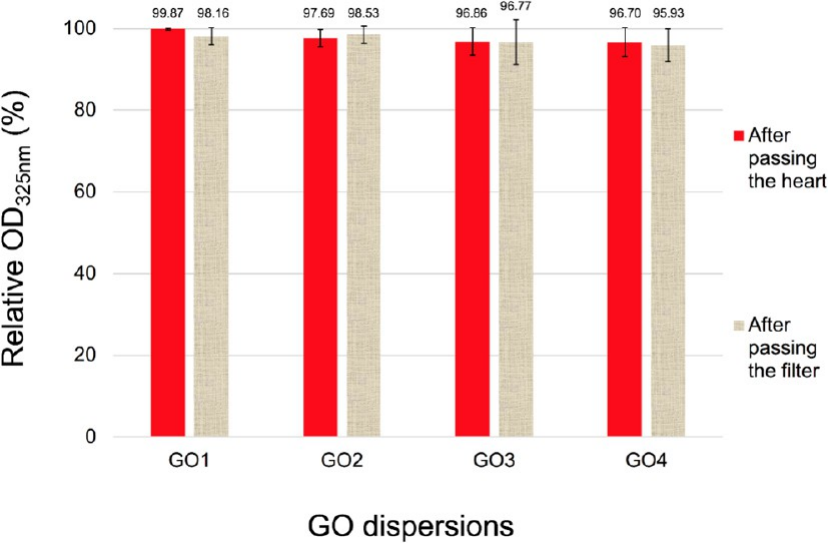
Changes in the relative optical density (OD_325_) (%) of randomly selected samples of GO dispersions (10 μg mL^–1^) after passing the heart (red) the 5 μm filter (canvas-like).

The GO stock dispersions were diluted to make the concentrations even and then sonicated to prevent coronary vessel obstruction, to reduce the polydispersity, and to increase the dispersion stability^54−56^ (Table S1 and Figure S1) by increasing the edge length of the GO particles, and possibly also by increasing the oxygen content.^56,57^ The sonication preceded the next step, *i.e.*, BSA-GO complexing, to avoid the protein surface function modification.^58^ Our protocol unified the particle sizes, reduced PDI, and markedly improved the dispersion stabilities in all types of tested GOs. Those observations were confirmed by spectrophotometric analysis performed to assess the relative number of particles that could be retained in the heart and at the 5 μm filter (Figure 2).

Both filter and hearts have retained only small amounts of GO particles, although more GO3 and GO4 than GO1 and GO2 particles were stopped both at the filter and in the perfused hearts. Importantly, those results positively verified the thesis of mainly the intravascular interaction of the particles with the heart tissues and effective as well as safe coronary vessel perfusion with the tested GO dispersions.

BSA, as a prominent component of blood plasma was used as a stabilizer in order to improve the overall GO wettability,^59^ and to reduce the physical interaction between the individual particles in the dispersion. Interactions of BSA with GO dispersions have already been analyzed.^29,59−61^ The description of the BSA-GO molecular interactions is reported in the SI. In our study, BSA concentrations—lower than those observed in blood—markedly improved GO dispersion stability under highly demanding conditions of isotonic solution containing calcium, magnesium, and sodium cations at high concentrations.

In this study, to the best of our knowledge, the BSA-stabilized isotonic aqueous GO dispersions were intentionally used for the first time in the direct perfusion of the mammalian heart. Nonetheless, GO pretreatment was used in ischemia-reperfusion study protocol,^62^ which led to an improvement in the coronary flow and reduction of the heart damage through free-radical scavenging mechanisms.

**Figure 3:**
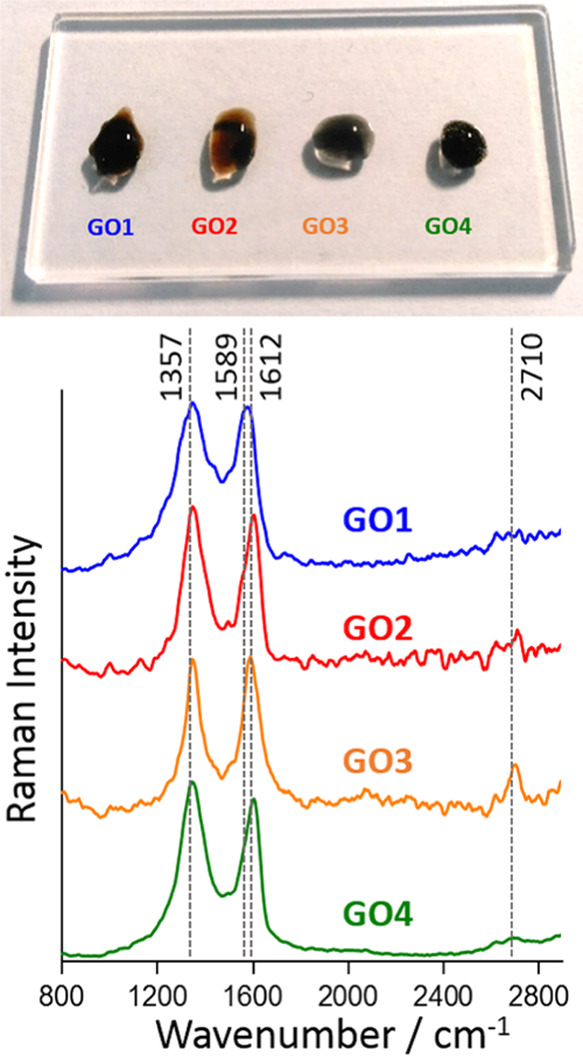
Raman spectra of the four GO dispersion samples at the initial concentrations (see the Experimental Section for details) in the fingerprint range; the inset shows a photograph of the samples.

To describe the GO particles and to further analyze the GO-BSA distribution in the heart, Raman spectroscopy was used. To examine the presence of GO in heart tissues at the cellular level, the five samples were analyzed after performing the following experiments: G1–10, G2–10, G3–10, G3–10 without the 5 μm KH filter, and G4–10. The results were compared with the Raman spectra of the four GO stock aqueous dispersions. Raman spectra of the standard samples (GO1–4) (Figure 3) show characteristic D- and G-bands in the CA (ca. 1357 cm^–1^ and in the range of from ca. 1589 to ca. 1612 cm^–1^, respectively).

The D-signal is assigned to the breathing modes of κ-point phonons of A_1g_ symmetry (describes the degree of carbon atom network disruption; it is low in pristine graphene) and the G-band to the E_2g_ phonons of sp^2^-carbon atoms. All dispersions differed significantly from one another in the analyzed particle population parameters (DLS). Generally, the most critical diagnostic information on the morphology and surface physicochemistry derives from the D-band to G-band intensity ratio (*I*_D_/*I*_G_ ratio). This parameter corresponds to a total number of structural and crystallographic defects with respect to the pure, *i.e.*, all-sp^2^-carbon graphene sheet.^63^ Those defects cover, to identify the most important ones, local distortion/waviness and all structural rearrangements, of various complexities, in the graphene hexagonal lattice such as (a) structural vacancies/gaps of a range of surfaces, *e.g.*, Stone–Wales defects, (b) grain boundaries, (c) sharp-ended edges, (d) sp^3^-hybridized carbon atoms (with a portion of sp^2^ ones) bearing functionalities such as epoxy (>O) (only sp^3^), hydroxyl (−OH) (mainly sp^3^), carboxyl (−COOH), and sulfo (−SO_3_H) groups (naming the most frequently abundant ones), *etc.*(64) Here, the lowest *I*_D_/*I*_G_ ratio recorded for GO3 sample (0.99) could be assigned to the least oxidized and hence most graphitized sample.^65^ And indeed, due to the entropic factor, GO3 predominantly formed spheroidal particles due to the presence of mostly edge, hydrogen-bonding flakes. At the same time, *I*_D_/*I*_G_ ratios for the other samples (GO1 = 1.04, GO2 = 1.06, GO4 = 1.11), due to the above-mentioned complexity, do not allow for the more specific characterizations as solely based on the Raman spectra.^65^

**Figure 4:**
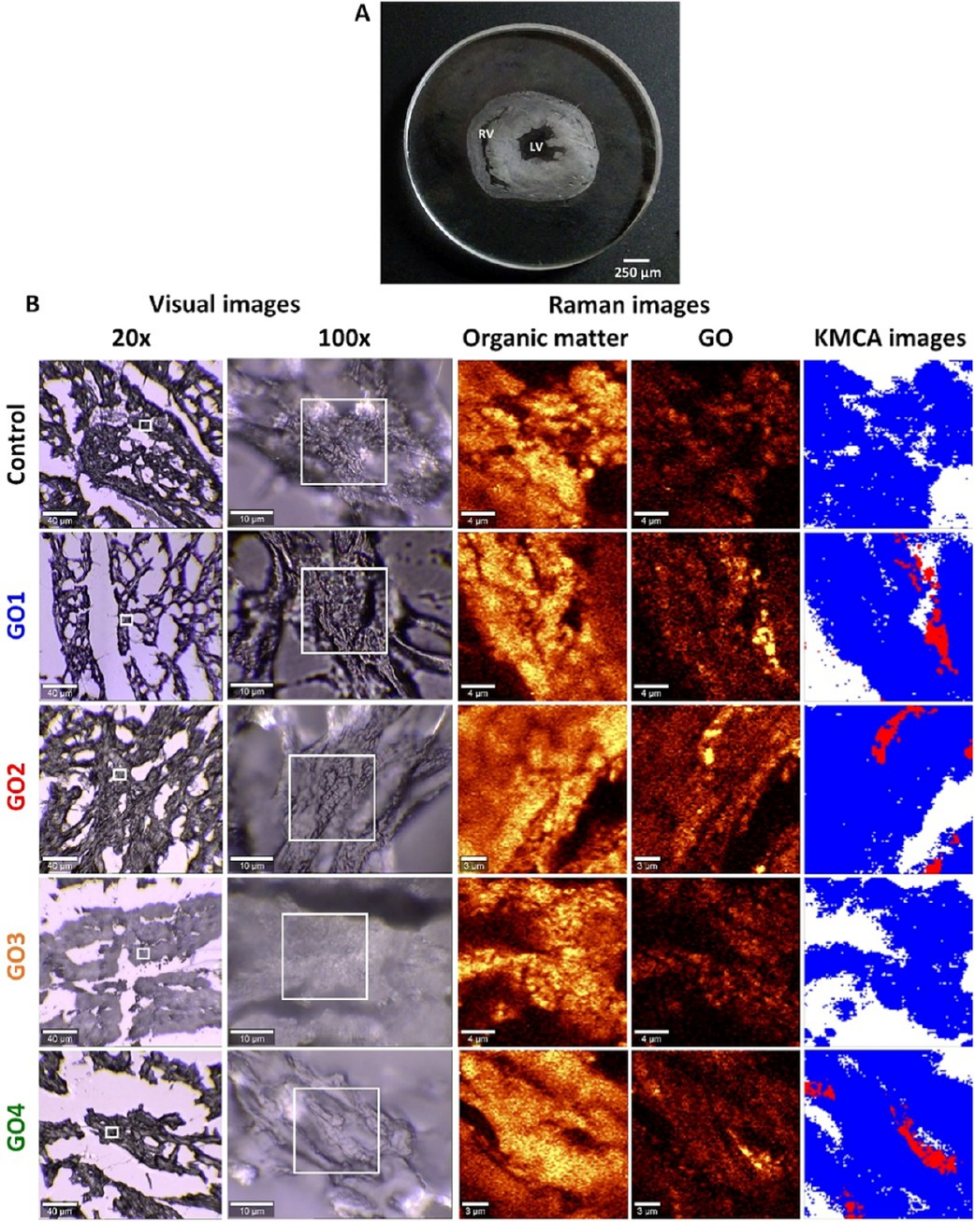
Raman spectroscopy analysis of perfused heart samples w/and w/o GO (study groups and control, respectively). (A) Representative heart slice image, (B) Representative optical images (20× and 100× magnification), Raman distribution images of the organic matter and GO in the tissue (obtained by integration of the signals in the ranges of 3030–2830 and 1657–1538 cm^–1^, respectively), and *k*-means CA (KMCA) images showing classes assigned to GO (red), tissue (blue), and background (no signal, white).

The Raman, CA, and micrographs representative for the heart tissue structure and the distribution of different GO types in the rat heart samples are given in Figures 4 and S2–S9. As shown, GO was found in the hearts perfused with GO1, GO2, and GO4 dispersions while, as expected, it was not detected in the control (*i.e.*, tissues not perfused with GO). Additionally, GO was not detected in the hearts of rats perfused with GO3 dispersion, irrespective of whether GO was filtered or not. Based on the comparison of the visual and Raman images, it was presumed that GO was distributed not only in the blood vessels but also in the surrounding tissues. The size of the GO clusters varied between 0.3 × 0.3 and 12.5 × 9.2 μm, and was independent of the size of GO nanoparticles. Nevertheless, no areas with the preferential localization of GO in the heart could be indicated based on the Raman spectroscopy analysis.

**Figure 5:**
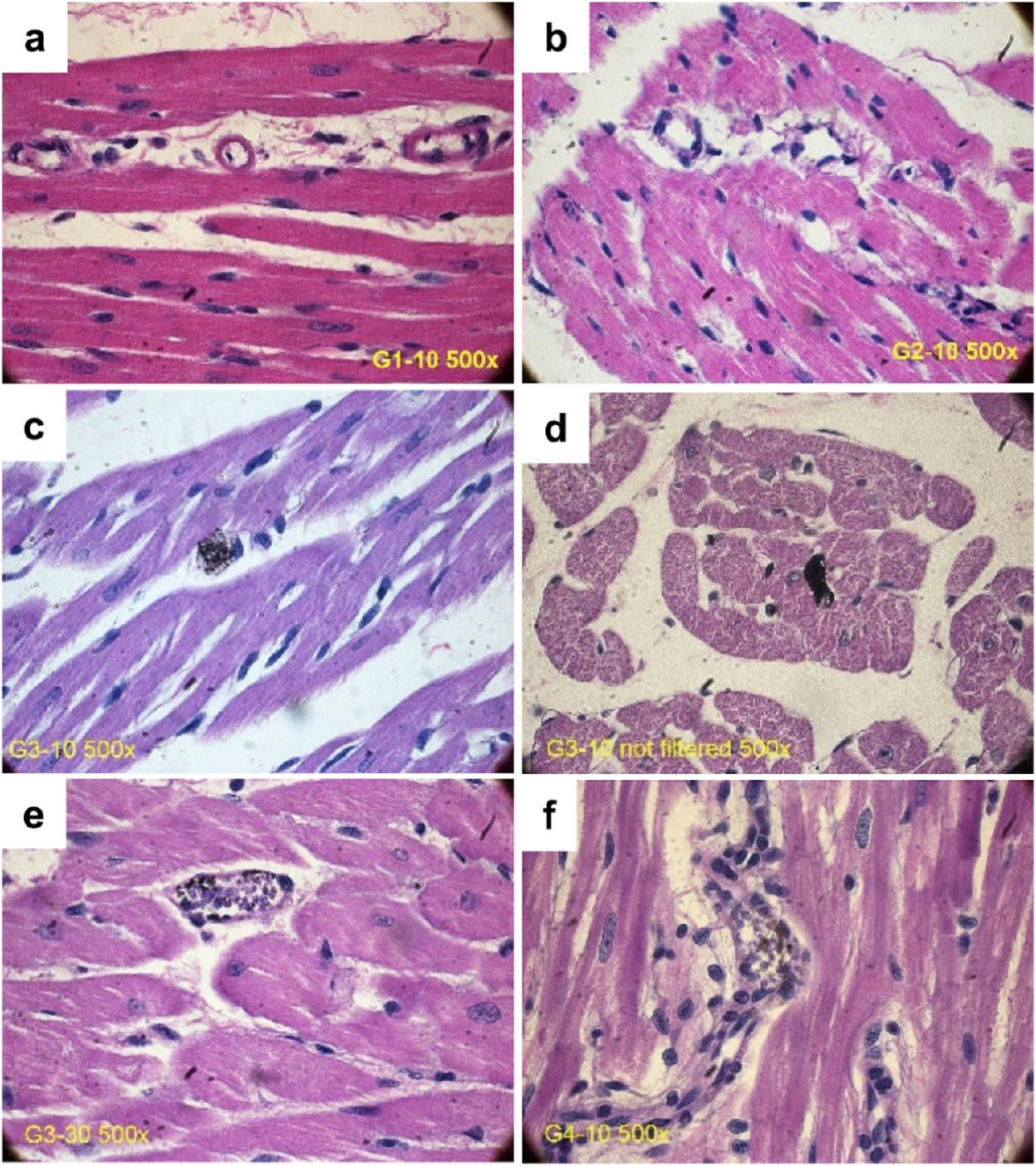
Representative histological assessment of heart samples after perfusion with GO1 10 μg mL^–1^ (a), GO2 10 μg mL^–1^ (b), GO3 10 μg mL^–1^ (c), GO3 10 μg mL^–1^ not filtered (d), GO3 30 μg mL^–1^ (e), and GO4 (10 μg mL^–1^) (f); standard staining.

Additionally, optical microscope analysis performed after standard hematoxylin and eosin staining revealed GO aggregates in a few vascular areas, mainly in GO3 and GO4 perfused hearts, without any features of cellular damage (Figure 5).

The agglomerated particles were seen in G3–10, G3–30, and G4–10, but not in G1–10 or G2–10 samples whose particles were thinner and more transparent. All of the particles were seen in rare microvessel blockages, but not in the extravascular tissues. The G3–30 samples emerged as the most condensed, black GO agglomerates. In turn, cellular structure damage was not observed in any group in the optical microscope analysis.

### Echocardiography Results

Echocardiography was used to analyze the diameters of the left ventricle and thickness of the left ventricular wall in two related rat strains. Left ventricular ejection fraction was high in all rats in echocardiography (mean 79.77% in Wistar rats and 82.75% in SHRs; nonsignificant (ns)). In our study, the mean body weight of a rat was significantly lower for SHRs, in spite of the longer pre-experimental observation with food access *ad libitum*. The observed mean interventricular septum diameter (IVS) systolic and diastolic thickness was slightly higher (ns) in all SHR rats compared to the Wistar control group, and the diameters of the left ventricle—both systolic and diastolic—were smaller in SHRs (ns). Significant differences in IVS in diastole (IVSd) were noted between Wistar and SHRs in favor of SHRs when animals were grouped according to the highest IVSd and IVSs parameters (Table S2). However, the interventricular septum systolic diameters (IVSs) were higher in the Wistar group (tendency, ns), which might suggest a stronger systolic thickness increase in the Wistar group. The important finding was a higher left ventricle internal diameter in diastole (LVIDd) in Wistar rats, which suggested a higher left ventricular diastolic volume. Taken together with a good contractility of the left ventricle (high LVEF), a higher stroke volume could be expected in the Wistar group (mainly due to higher body mass). This finding, together with nonsignificant differences in left ventricular diameters and ventricular wall thickness between the rat strains (calculated for all animals in the compared strains, Table S2), supports the hypothesis of stronger left ventricular hypertrophy in SHRs.

### Heart Perfusion

#### GO and the Coronary Flow

The cardiac effects of GO, among its 4 forms, were not uniform. The mean coronary flow (CFM) observed in the working heart was transiently reduced with GO1 (single-to-few-layer, highly oxidized) in normotensive hearts (10 μg mL^–1^, *Z*-Ave 710 nm) (Table 2), whereas in hypertensive animals, there was no change in CFM (GO1, 10 μg mL^–1^, *Z*-ave 420 nm) (Table 3). GO2 (predominantly single-layer, high O-content) has lowered the CFM in normotensive (10 μg mL^–1^, *Z*-Ave 601 nm) and hypertensive animals (Tables 2 and 3) (10 μg mL^–1^, *Z*-Ave 400 nm), but no significant change was observed at higher GO concentration in healthy animals (30 μg mL^–1^, *Z*-Ave 450 nm) (Table S3). GO3 (granular, lower O-conc.) caused no changes of CFM in the normotensive rats (10 μg mL^–1^, *Z*-Ave 410 nm), even though the concentration was increased (30 μg mL^–1^, *Z*-Ave 360 nm), but significantly lowered the CFM in hypertensive rats (10 μg mL^–1^, *Z*-Ave 460 nm) (Tables 2, 3, and S4). GO4 (lower O-conc., containing NH_4_^+^ groups) has lowered the CFM in normotensive rats (10 μg mL^–1^, *Z*-Ave 477.80 nm), while a tendency to a lower CFM was observed in hypertensive rats (10 μg mL^–1^, *Z*-Ave 550 nm, Tables 2 and 3).

**Table 2:** Basic Cardiac Parameters in the Groups of Wistar Rats Perfused with GO1-4 (10 μg mL^–1^) in the Working Heart Mode^a^,^b^

parameter	group	control	GO perfusion start	GO perfusion half-time	GO perfusion end
	exp. stage	cwh1–2	twh2–0	twh2–1	twh2–2
CFM coronary flow mean (mL min^–1^)	G1–10	22 ± 3	21 ± 3^c^	19 ± 2^c^	21 ± 7
	G2–10	19 ± 6	17 ± 3	14 ± 4	11 ± 5^d^
	G3–10	19 ± 5	19 ± 7	15 ± 8	15 ± 6
	G4–10	17 ± 6	17 ± 7	14 ± 7^c^	12 ± 8^c^,^d^
AoFM aortic flow mean (mL min^–1^)	G1–10	50 ± 7	35 ± 9^c^	36 ± 15^c^	41 ± 9
	G2–10	39 ± 13	39 ± 16	40 ± 17	39 ± 14
	G3–10	42 ± 12	39 ± 12	43 ± 17	31 ± 22
	G4–10	45 ± 8	42 ± 8	45 ± 11	38 ± 16
HRAP heart rate from aortic pressure (1 min^–1^)	G1–10	213 ± 28	227 ± 31	233 ± 45	250 ± 23
	G2–10	231 ± 49	241 ± 14	232 ± 41	213 ± 50
	G3–10	230 ± 37	234 ± 11	233 ± 11	232 ± 25
	G4–10	238 ± 28	248 ± 18	246 ± 16	246 ± 21
APM aortic pressure mean (mmHg)	G1–10	92 ± 11	89 ± 10^c^	88 ± 11^c^	89 ± 10
	G2–10	91 ± 8	91 ± 9	90 ± 9	87 ± 8^c^
	G3–10	91 ± 6	92 ± 4	90 ± 3	85 ± 7
	G4–10	88 ± 4	89 ± 4	88 ± 4	85 ± 7

a Study groups: G1-10, G2-10, G3-10, and G4-10.

b Abbreviations: exp stage, experimental stage.

c *p* < 0.05 with cwh1–2.

d *p* < 0.05 with twh2–0.

**Table 3:** Basic Cardiac Parameters in the Groups of SHRs Perfused with GO1-4 (10 μg mL^–1^) in the Working Heart Mode^a^,^b^

parameter	group	control	GO perfusion start	GO perfusion half-time	GO perfusion end
	exp. stage	cwh1–2	twh2–0	twh2–1	twh2–2
CFM coronary flow mean (mL min^–1^)	G1–10SHR	13 ± 2	12 ± 2	13 ± 3	11 ± 3
	G2–10SHR	14 ± 4	12 ± 4^c^	14 ± 3	12 ± 5^c^
	G3–10SHR	13 ± 4	12 ± 4	10 ± 5^c^	10 ± 4^c^,^d^
	G4–10SHR	12 ± 3	12 ± 2	11 ± 3^c^	10 ± 4
AoFM aortic flow mean (mL min^–1^)	G1–10SHR	32 ± 7	32 ± 6	32 ± 9	32 ± 10
	G2–10SHR	31 ± 10	32 ± 11	31 ± 9	32 ± 12
	G3–10SHR	33 ± 8	33 ± 7	27 ± 7^c^	25 ± 8^c^,^d^
	G4–10SHR	38 ± 5	36 ± 6^c^	34 ± 6^c^	32 ± 6^c^
HRAP heart rate from aortic pressure (1 min^–1^)	G1–10SHR	196 ± 24	195 ± 22	206 ± 19	188 ± 15
	G2–10SHR	157 ± 42	156 ± 51	177 ± 65	158 ± 53
	G3–10SHR	208 ± 39	212 ± 38	199 ± 35	188 ± 26^d^
	G4–10SHR	206 ± 40	203 ± 23	186 ± 17	176 ± 23^c^
APM aortic pressure mean (mmHg)	G1–10SHR	88 ± 4	88 ± 3	89 ± 4	86 ± 3
	G2–10SHR	84 ± 5	86 ± 7	86 ± 6^c^	83 ± 6
	G3–10SHR	86 ± 4	86 ± 4	83 ± 3	83 ± 5^c^,^d^
	G4–10SHR	88 ± 2	88 ± 2	87 ± 2^c^	85 ± 2^c^

a Study groups: G1-10SHR, G2-10SHR, G3-10SHR, and G4-10SHR.

b Abbreviations: exp stage, experimental stage.

c *p* < 0.05 with cwh1–2.

d *p* < 0.05 with twh2–0.

In the Langendorff perfusion under the constant pressure, when the hearts were still contracting freely with the empty chambers, the situation was different: GO1 and GO2 reduced the CFM significantly both in normotensive (GO1: 10 μg mL^–1^, *Z*-Ave 710 nm; GO2: 10 μg mL^–1^, *Z*-Ave 600 nm) and hypertensive rats (GO1: 10 μg mL^–1^, *Z*-Ave 420 nm, GO2: 10 μg mL^–1^, *Z*-Ave 400 nm) (Tables 4 and 5); GO2 induced a significant CFM reduction also at higher GO concentration in the normotensive rats (30 μg mL^–1^, 450 nm, Tables 4–6). GO3 caused no deterioration of CFM in none of the tested groups (10 μg mL^–1^: normotensive 410 nm; hypertensive 460 nm; normotensive at high GO3 concentration 30 μg mL^–1^, 360 nm, Tables 4, 5, and 6) when it was administered into coronary perfusion in the Langendorff preparation. In GO4 Langendorff perfusion, in normotensive rats’ group, the significant deterioration of CFM was observed (10 μg mL^–1^, *Z*-Ave 480 nm, Table 4), whereas in the hypertensive rats, there was no CFM decrease (10 μg mL^–1^, *Z*-Ave 550 nm) (Table 5).

**Table 4:** Bradykinin-Stimulated Coronary Vessel Reactivity Observed as the Coronary Flow Increase (Bk) in Control Perfusion (Con) and in Graphene Oxide Perfusion (Graphene) Stages during Constant-Pressure Langendorff Perfusion in Groups of Normotensive Wistar Rats^a^

coronary flow mean CFM (mL min^–1^)	before Bk control	Bk	after Bk	Bk, % of control	after Bk, % of control
con G1–10	14 ± 1^*c*^	19 ± 2^*b*^	16 ± 2	135 ± 21	113 ± 7^*j,k*^
graphene G1–10	10 ± 1^*c*^	18 ± 3^*b*^	15 ± 1^*b*^	173 ± 48	142 ± 27^*l*^
con G2–10	10 ± 4^*d*^	16 ± 6^*b*^	13 ± 3^*b*^	153 ± 43	127 ± 25^*j*^
graphene G2–10	6 ± 3^*d*^	16 ± 5^*b*^	10 ± 4^*b*^	251 ± 79^*h,I*^	153 ± 27^*m,n*^
con G3–10	13 ± 5	19 ± 6^*b,f*^	15 ± 5	163 ± 60	122 ± 32^*k*^
graphene G3–10	11 ± 5	15 ± 6^*f*^	13 ± 6	141 ± 30^*h*^	119 ± 17^*m*^
con G4–10	12 ± 4^*e*^	16 ± 4^*b,g*^	14 ± 4	137 ± 23	116 ± 14
graphene G4–10	7 ± 3^*e*^	10 ± 5^*b,g*^	8 ± 5	141 ± 36^*i*^	93 ± 41^*l,n*^

a Abbreviations: CFM, coronary flow mean. Bk, bradykinin. The first column represents the stages of the experiment: Con—control stage (no GO in the KH solution), Graphene—test stage with GO in KH solution; Before Bk: the initial stage during Langendorff perfusion stages in lang1–cl1 and lang2–cl2, see Figure 1) used as control before Bk infusion; Bk: bradykinin infusion periods with GO (bl2) and without GO (Con–bl1); after Bk: post bradykinin infusion periods in Langendorff stages (bl1stop and bl2stop in Figure 1) Bk% of control: relative increase of CFM – value of BK stage as a percent of the value of the control stage in the same line; after Bk% of control: relative value of mean coronary flow in comparison with before Bk control stages (the same calculation as in the Bk% of control). ^*b*^ <0.05 with control in the same line. ^*c,d,e,f,g,h,i,j,k,l,m,n*^*p* < 0.05 only between the same superscripts.

**Table 5:** Bradykinin-Stimulated Coronary Vessel Reactivity Observed as the Coronary Flow Increase (Bk) in Control Perfusion (Con) and in Graphene Oxide Perfusion (Graphene) Stages during Constant-Pressure Langendorff Perfusion in G1, G2, G3, G4-10SHR Groups of Hypertensive SHR Rats^a^

coronary flow mean CFM (mL min^–1^)	before Bk control	Bk	after Bk	Bk, % of control	after Bk, % of control
con G1–10SHR	8 ± 1^*c*^	13 ± 2^*b*^	9 ± 1	157 ± 20^*f,g*^	107 ± 7
graphene G1–10SHR	6 ± 1^*c*^	13 ± 3^*b*^	9 ± 2^*b*^	188 ± 12^*h,i*^	131.14 ± 10^*c*^
con G2–10SHR	10.6 ± 1.9^*d*^	13 ± 1^*b*^	11 ± 1	131 ± 19^*f*^	105 ± 11
graphene G2–10SHR	8.2 ± 1.3^*d*^	13 ± 1^*b*^	9 ± 1^*b*^	171 ± 33^*j*^	119 ± 15^*c*^
con G3–10SHR	8.6 ± 3.6	10 ± 3^*b*^	9 ± 3	126 ± 17^*g,k*^	105 ± 10
graphene G3–10SHR	8.1 ± 3.6	11 ± 3^*b*^	7 ± 2	141 ± 23^*h*^	98 ± 22^*c*^
con G4–10SHR	7.6 ± 1.6	11 ± 1^*b,e*^	8 ± 1	157 ± 41^*k*^	110 ± 29
graphene G4–10SHR	7.3 ± 1.3	8 ± 2^*e*^	7 ± 1	120 ± 30^*i,j*^	96 ± 14^*c*^

a Abbreviations: See Table 4. ^*b*^ <0.05 with control, ^*c,d,e,f,g,h,i,j,k*^*p* < 0.05 with the same superscript.

**Table 6:** Bradykinin-Stimulated Coronary Vessel Reactivity Observed as the Coronary Flow Increase (Bk) in Control Perfusion (Con) and in Graphene Oxide Perfusion (Graphene) Stages during Constant-Pressure Langendorff Perfusion in Groups of Normotensive Wistar Rats Perfused with an Increased (30 mg L^–1^) Concentration of the GO2 and GO3^a^

coronary flow mean CFM (mL min^–1^)	before Bk control	Bk	after Bk	Bk, % of control	after Bk, % of control
con G2–30	12 ± 3	17 ± 5^*b*^	11 ± 3	150 ± 37	96 ± 10
graphene G2–30	10 ± 2	16 ± 4^*b*^	11 ± 3	171 ± 30^*d*^	118 ± 21
con G3–30	13 ± 3	18 ± 3^*b,c*^	14 ± 3	140 ± 16	108 ± 15
graphene G3–30	13 ± 4	14 ± 3^*c*^	13 ± 3	103 ± 12^*d*^	102 ± 15

a Abbreviations: See Table 4. ^*b*^ <0.05 with control in the same line. ^*c,d*^*p* < 0.05 with the same superscript.

#### GO and Reaction to Bradykinin

During bradykinin infusion in the GO1 and GO2 groups, both in the normotensive and hypertensive hearts, a significant coronary flow increase was observed both in control and in the GO1, GO2 (10 μg mL^–1^) perfusion stages (Tables 4 and 5). This effect was still significant after the cessation of bradykinin administration (GO1, GO2; Tables 4, 5, “after Bk” column). For the increased concentration of GO2 (30 μg mL^–1^), a significant bradykinin-induced increase of the CFM was also observed, but it was not prolonged beyond the drug infusion period (Table 6). In the GO3 groups, the reactions to infused bradykinin were more variable. In all control perfusion periods, a significant increase in CFM was seen as a response to bradykinin. When bradykinin with tested GO3 (10 μg mL^–1^) was infused in the normotensive group, a nonsignificant increase in CFM was observed (Table 4). In the hypertensive hearts, the stronger response to bradykinin was observed: an increase in CFM was significant during the drug and GO3 infusion (Table 5). When a higher GO3 concentration (30 μg mL^–1^) was used in normotensive hearts, no increase in CFM was observed during GO3 and bradykinin administration. Moreover, in the G3–30 group, CFM during the bradykinin infusion was significantly lower than in the control perfusion stage (Table 6). In the GO4 normotensive group, during the perfusion of the hearts with GO4 (10 μg mL^–1^) and bradykinin, a significant increase in CFM was observed. At the same time, the CFM increase in this stage (GO4, 10 μg mL^–1^ Lang 2, see Figure 1, and graphene G4–10, see Table 4) was weaker and CFM was significantly lower than in the control perfusion stage without the GO4 (GO4, 10 μg mL^–1^, Lang 1, see Figure 1 and con G4-10, see Table 4). In the hypertensive hearts, no response to bradykinin was noted during the GO4 perfusion, and CFM was significantly lower in the GO4-bradykinin perfusion stage than in the control bradykinin perfusion period (Table 5).

#### GOs and the Main Cardiac Parameters

The main cardiac parameters monitored in this study were CFM (reported above), mean aortic flow (AoFM), heart rate (HRAP), and mean aortic pressure (APM). HRAP was calculated from the aortic pressure trace and observed during the working heart perfusion periods. GO1 had no significant influence on HR, in either normotensive or hypertensive hearts. In the normotensive hearts, in the G1–10 group, a subtle tendency to an increase in HR during perfusion with GO was noticed (Table 2). In none of the GO2 groups, a change in HR could be observed during the GO2 perfusion periods (Tables 2, 3, and S3). HR in GO3 groups was not changed significantly in normotensive hearts, either at low or high concentrations of GO3. On the contrary, in the hypertensive hearts, HR was significantly lowered (Tables 2, 3, and S4). The normotensive hearts perfused with GO4 presented a stable HR, with a slight tendency to increase. The hypertensive hearts treated with GO4 dispersion significantly lowered HR upon the perfusion. APM was not changed with the GO1 perfusion in any of the groups (Tables 2 and 3), whereas GO2 significantly lowered APM in normotensive hearts, both in low and high doses (Tables 2 and S3). On the contrary, in the hypertensive hearts, no changes in APM were observed (for GO2). APM in GO3 groups presented a significant decrease in the hypertensive group, whereas in both normotensive GO3 groups (G3–10 and G3–30), no significant APM deterioration was observed (Tables 2, 3, and 6). The hypertensive hearts perfused with GO4 presented a significantly lower APM during the GO4 perfusion period, whereas for the normotensive hearts, no APM deterioration was observed (Tables 2 and 3).

The aortic flow is the most important cardiac parameter describing the dynamic aspect of cardiac function in the working heart protocol. Its value was influenced by the differences in body weight (b.w.) between the SHR and Wistar rats. AoFM in GO1 groups showed no significant changes, either in normotensive or in hypertensive hearts. Precisely, in the normotensive group, a momentary significant decrease of AoFM was noted, but at the end of the GO1 perfusion period, no significant AoFM deterioration was noted (Tables 2 and 3). In the GO2 groups, no significant AoFM deterioration was observed, although in the G2–30 group, the tendency to lower AoFM could be noticed (Tables 2, 3, and S3). The significantly lowered AoFM was noted in the GO3 perfusion period in the hypertensive hearts. In the normotensive hearts, no significant deterioration was observed (Tables 2, 3, and S4). In hearts perfused with GO4, a significant deterioration of the AoFM was seen in the hypertensive hearts, whereas only a nonsignificant tendency to lower AoFM was found in the normotensive group (Tables 2 and 3).

#### AFM Studies

**Figure 6:**
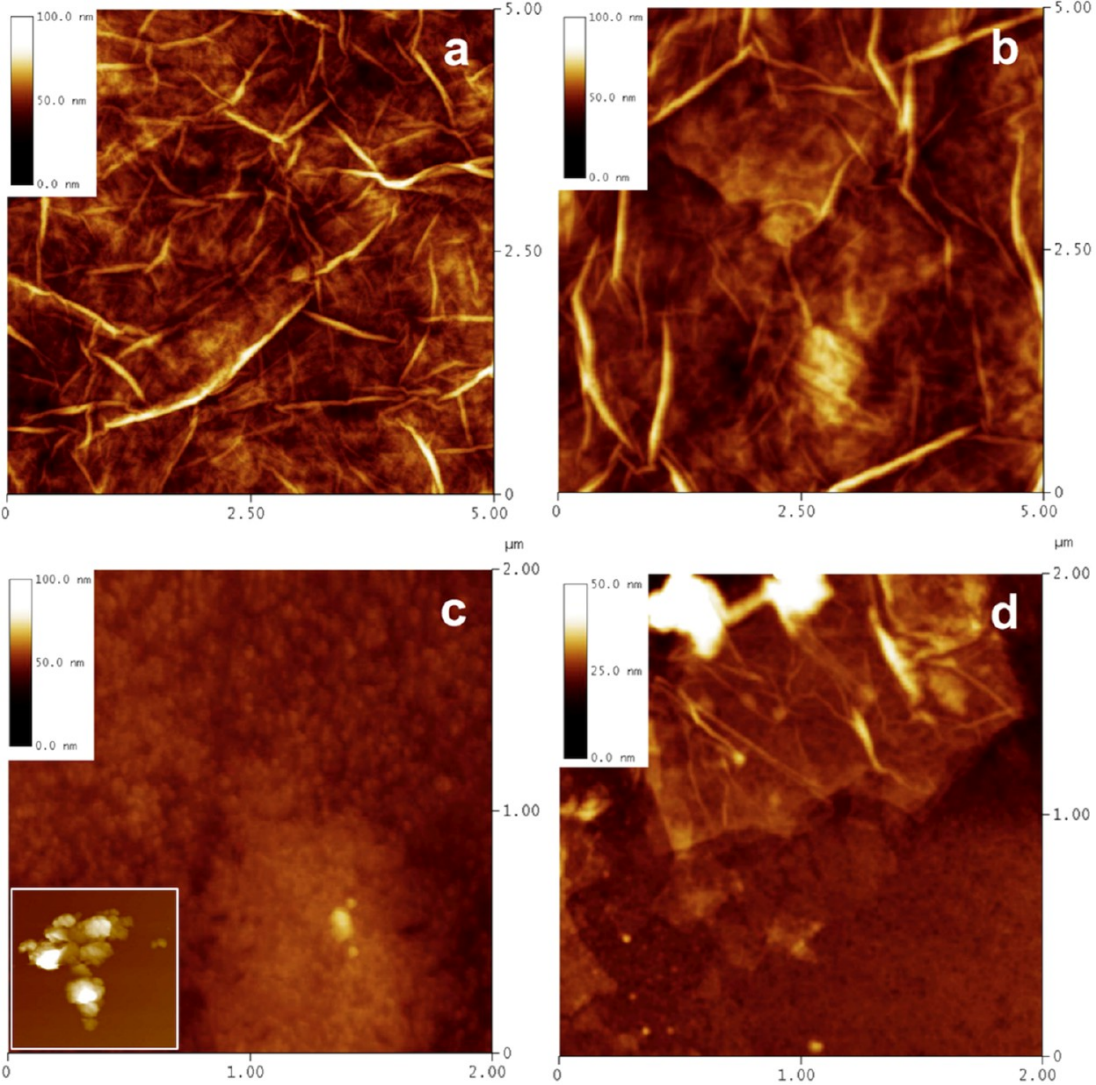
AFM images of GOs in the original stock: GO1 (a), GO2 (b), GO3 (c) with an inset representing one of the observed complex structures, and GO4 (d).

AFM images of GOs in the original stock are given in Figure 6.

Strikingly, otherwise than for the particle shapes of GO1, GO2, and GO4, GO3 flakes appeared granular. All of the other GOs were found to be laminar, with a variable number of layers but similar number of wrinkles per area unit and a comparable height of the wrinkles. At the consecutive stages of the preparation protocol the picture, the shape of the edges, also shape and size of the particles, and number of layers were changing.

#### Dosage and Toxicology of GOs

The effective doses of the GO in our study were calculated from the AoFM and CFM. Using the applied perfusion study protocol, we can differentiate the heart dose that has passed directly and only through the coronary vessels (CFM) separately from the dose of the GO that passed through the heart chambers and the coronary vessels (for the latter: the body dose is calculated from the cardiac output that is the result of the addition of CFM and AoFM). It refers to the total dose for the animal *in vivo*. The SHRs accepted, directly to the heart muscle, the dose ranging from 7.74 to 9.42 mg kg^–1^ b.w., whereas the total body dose ranged from 23.71 to 26.84 mg kg^–1^ b.w. In the Wistar normotensive rats, the heart doses were between 7.00 and 9.78 mg kg^–1^ b.w., while the mean body dose was between 20.67 and 26.44 mg kg^–1^ b.w. In the groups with the increased GOs concentration (G2–30 and G3–30), the heart doses were 21.67 mg kg^–1^ b.w. (G2–30) and 25.67 mg kg^–1^ b.w. (G3–30), whereas the total body doses were 58.67 and 62.67 mg kg^–1^ b.w. for G2–30 and G3–30, respectively. The comparison of the GO quantities retained in the heart is shown in Figures 2 and 5. GO has been tested in oral, inhalation, intraperitoneal, and intravenous administration routes (see the SI). The dose-dependent effects of GO administered are presented in the SI (SI15).

It is challenging to discuss our results with the literature because the cardiac effects of GO administration are very rare and conflicting. In the study by Zhang et al.,^66^ the effects of: (a) GO (thickness 3–4 nm and lateral size 100–200 nm), and (b) reduced (to a variable degree) GO (rGO), in rat cardiomyocyte culture were tested. In the cited study, GO and rGO (proportionally to a reduction degree) increased the cellular ROS content and lowered the mitochondrial membrane potential, suggesting the mitochondrial origin of at least a part of the ROS level increase that was observed. Recently, the cardioprotective effects of the 10–15-layer GO and oxidized (C/O ratio of 2.5–2.6) with a flake size between 0.1 and 200 μm were reported by Voitko et al.^62^ In their data, the post-ischemic CFM significantly improved in the GO-pretreated group (a 32% increase in comparison with the control). The authors proposed that the protective effect could be mediated through an ROS scavenging mechanism and demonstrated its chemical origin. The effectiveness of the low GO concentration in the *in vitro* entrapment of superoxide radicals is an important finding.

The above reports are also important in our study. The bradykinin test (with an indomethacin blocking the prostacyclin synthesis) was used as the tool to assess the NO-dependent coronary vasodilatation in the presence and absence of GO. It is well known that NO is a free-radical individual and, hence, acts as a ROS. Bradykinin with GO1 and GO2 induced a significant CFM increase that was considerably higher than the control, even after stopping the bradykinin infusion (Tables 4 and 5). The increase of the GO2 concentration (G2–30) caused shortening of the observed bradykinin effect to the bradykinin infusion period only, but not beyond (Table 6). Low-oxygen-GO3 with bradykinin has induced a significant CFM increase in NO-deficient SHRs, not significant increase of CFM in normotensive Wistars (Tables 4 and 5). At the same time, at the higher concentration, it hampered an increase of CFM in the Wistar rats (Table 6). In GO4 groups, Wistar rats displayed a significant increase of CFM—both in the control and GO perfusion periods, but the absolute CFM values were significantly lower in the GO4 perfusion than in the control period (Table 4). In the SHR group, the NH_4_^+^-containing, low-oxygen-content GO4 stopped the increase of the CFM during the bradykinin infusion and the absolute CFM values were significantly lower than in the control period (Table 5). Those observations suggest more intensive quenching of the expected increased NO concentration during the bradykinin infusion at higher concentrations of tested GOs. Moreover, at least two types of reactions to bradykinin with low-oxygen GO could be observed in SHRs. The differences between the GO3 and GO4 effects on SHRs in bradykinin tests suggest the importance of oxygen content in the particle structure. GO3 and GO4 both revealed a similar defect of the carbon network structure in Raman spectroscopy, but the GO4 contained less oxygen with additional NH_4_^+^ in “defected” positions, which may have had the hampering effect on the bradykinin-induced CFM increase in hypertensive rats in contrast to GO3 which was stimulating the CFM increase.

The bradykinin tests confirmed that the GO particles modified the response of the heart endothelium to various extents, depending on the GO structure. In this study, we have demonstrated that oxygen-rich and single-to-few-layer (*i.e.*, below 10) GOs did not hamper the endothelial response to bradykinin, but prolonged its effect beyond the drug infusion time (at low concentration) (Tables 4 and 5). On the other hand, GOs with a lower oxygen content, in a granular form, or bearing surface NH_4_^+^ groups, reduce the NO-mediated coronary vasorelaxation. The antibradykinin (or NO scavenging) effect of GO was seen in G2–30, G3–30, and G4–10SHR, and was the weakest in G2–30 and the strongest in G4–10SHR.

In summary, for the main cardiac parameters, the GO1 effects on Wistar rats cardiac function were transient. The initial reduction of the CFM, AoFM, and APM was reversed at the end of observation period and these parameters became only not significantly lower than in the GO1 control period. In fact, the smallest decrease occurred in CFM. The reversal of the negative trend was in part dependent on the increase (ns) of the HRAP that was noted in the second part of the test. In contrast, in the smaller and hypertrophied SHR hearts, all of the observed working heart parameters were less reduced by GO1 and the changes were nonsignificant. In this group (G1–10SHR), HRAP displayed a subtle tendency to decrease at the end of the observation period (Table 3).

GO2 showed a different pattern of the changes in the Wistar heart parameters. In this group, CFM was decreasing constantly, reaching the significant level of decrease at the end of the observation period (Table 2). Moreover, the significant reduction of the APM was accompanied by a nonsignificant decrease of the HRAP and no change in AoFM. GO2 was also tested in an increased concentration of 30 μg mL^–1^ and no deterioration of the parameters was observed there (Table S3). Further, the SHR hearts perfused with GO2 presented an unstable reduction of CFM, but AoFM, HRAP, and APM were not changed (Table 3).

The initially granular GO3 in Wistars nonsignificantly reduced the CFM, AoFM, and APM, but not HRAP. In this group, all of the parameters presented the tendency to decrease with the smallest change in HRAP (Table 2). GO3 was tested also at the high concentration (30 μg mL^–1^) and no change in the heart parameters was noted (Table S4). The GO3 was harmful to the SHR hearts with a significant reduction in all of the measured cardiac parameters (Table 3).

The Wistar hearts perfused with GO4 presented a significant reduction of CFM, leading to only a slight lowering of AoFM and APM. In this group, similarly, HRAP showed the tendency to increase during the GO4 perfusion period (Table 2). GO4 in the SHR hearts emerged as harmful, exhibiting a significant reduction of all of the cardiac parameters, except from CFM. Here, CFM presented only the tendency to decrease at the end of the observation period (Table 3).

The perfusion tests in the Langendorff protocol were planned to analyze the changes of CFM under constant pressure. In this protocol, CFM was lower and less dependent on the heart contraction as the heart was not pumping actively. Here, the CFM decrease was observed for GO1, GO2, and GO4, but not for GO3 (Table 4). The observed difference between CFM in the control perfusion stage and GO perfusion periods was dependent mostly on an increase of the particle-induced flow resistance (Table 4, column “before Bk control” lines Con versus Graphene). In the GO3 perfusion groups, no CFM decrease was observed during the Langendorff perfusion. Indeed, in the initial, *i.e.*, pre-sonication stage of the experimental preparation, GO3 was the only granular form of GO among the studied GO variants.

## Conclusions

Our results confirm that the impact of GO on the heart depends on the morphology and surface physicochemistry of GO nanoparticles, varying between the normo- and hypertensive hearts. Specifically, the effects range from the increase in the coronary flow, through the inert effects of GO perfusion to the cardiodepressive effects. ROS scavenging effects, also previously reported, might be responsible for the observed reduction of the NO-dependent, bradykinin-stimulated coronary vasodilatation. This phenomenon was more evident in the experiments recorded for the low-oxygen GO forms and at higher concentrations of highly oxygenated GOs. At the same time, in the presence of GO, the hypertensive hearts, characterized by possible topological and functional defects of the endothelium, were deteriorating easier, especially in the case of low-oxygen-content GOs (GO3, GO4).

The pathomechanism of cardiac cellular damage observed *in vitro* was reported elsewhere to be originating from the changes in the mitochondrial membrane potential, and to be dose-dependent (at concentrations in the range of 10–100 μg mL^–1^).^46^ Here, it seems to be less important due to a low amount of GO retained in heart. Accordingly, no signs of the cellular damage were detected. Overall, this behavior, as shown in Table 3, would corroborate stable CFM accompanied by deterioration of the other cardiac parameters (observed in group G4–10SHR). This behavior can be tuned in therapies as the mitochondrion, being an important micromachinery in the heart failure, serves as the Adenosine triphosphate (ATP) generation site and the potential source of ROS and RNS, and a possible target for the antioxidant therapies.^38^ Apart from the above, one must clearly admit that the effect of increasing HR, recorded for the single-to-few-layer, high O-content GO, requires further analysis.

GO is frequently reported to have adequate biocompatibility, drug loading capacity and capability, and also several structural modification possibilities to become an effective drug transport platform.^4^ Drug release from GO complexes can be controlled with phototherapy from outside the body.^67^ All of these promising GO bioapplications can be made easier and safer with BSA, the natural stabilizer of the GO dispersions in the circulatory system.

## Supporting Information Available

Experimental data for the experimental methods; DLS studies; the experimental results from histopathology combined with Raman spectroscopy; statistics on the echocardiographic diameters of the Wistar and SHR hearts; effects of GO on cardiac parameters, and discussion on the GO circulatory and the main organ effects (PDF)
