# Supplementary material for: Graphene Oxide Significantly Modifies Cardiac Parameters and Coronary Endothelial Reactivity in Healthy and Hypertensive Rat Hearts Ex Vivo
Source: ACS Omega. 2024 Jun 18;9(26):28397–411. doi: 10.1021/acsomega.4c02291 (PMC11223131; doi:10.1021/acsomega.4c02291)
Supplement: Supplementary file 1 — ao4c02291_si_001.pdf [file ao4c02291_si_001.pdf]

Supplementary Information

for

Graphene Oxide Significantly Modifies Cardiac Parameters and Coronary Endothelial Reactivity in Healthy and Hypertensive Rat Hearts *Ex Vivo*

Marcin Z. Krasoń[a,b,*], Anna Paradowska[a], Sławomir Boncel[c,d,*], Mateusz Lejawa[a,e], Martyna Fronczek[a,e], Joanna Śliwka[a,b], Jerzy Nożyński[b], Piotr Bogus[a], Tomasz Hrapkowicz[b], Krzysztof Czamara[f], Agnieszka Kaczor[g], Marek W. Radomski[h]

[a] Silesian Park of Medical Technology Kardio-Med Silesia, Marii Skłodowskiej-Curie 10C, 41-800 Zabrze, Poland

[b] Department of Cardiac, Vascular and Endovascular Surgery and Transplantology, Silesian Center for Heart Disease, Medical University of Silesia in Katowice, Marii Skłodowskiej-Curie 9, 41-800 Zabrze, Poland

[c] Silesian University of Technology, Faculty of Chemistry, Department of Organic Chemistry, Bioorganic Chemistry and Biotechnology, Krzywoustego 4, 44-100 Gliwice, Poland

[d] Silesian University of Technology, Centre for Organic and Nanohybrid Electronics (CONE), Konarskiego 22B, 44-100 Gliwice, Poland

[e] Department of Pharmacology, Faculty of Medical Sciences in Zabrze, Medical University of Silesia in Katowice, Jordana 38, 41-808 Zabrze, Poland

[f] Jagiellonian Centre of Experimental Therapeutics (JCET), Jagiellonian University, M.Bobrzyńskiego 14, 30-348 Kraków, Poland

[g] Faculty of Chemistry, Jagiellonian University, Gronostajowa 2, 30-387 Kraków, Poland

[h] College of Medicine, University of Saskatchewan, Department of Anatomy, Physiology and Pharmacology, 107 Wiggins Rd, Saskatoon SKS7N 5E5, Canada

* Correspondence: kramarci22@gmail.com; slawomir.boncel@polsl.pl

METHODS

Preparation of GO dispersions

To prepare GO samples, stock GO dispersions were shaken several times, and the volumes of 1.4 mL (GO1), 1.75 mL (GO2), 7 mL (GO3), and 7 mL (GO4) were transferred to 75-mL-beakers, and dispersed to the final volume of up to 50 mL with ultrapure water (GO at a concentration of 140 µg mL^{-1}). Further, the beakers were placed in the water bath, cooled to 4 °C, and sonicated directly with the Ultrasonic Processor UP200St (Hielscher Ultrasonics GmbH, Teltow, Germany) using a sonotrode (S26d14) immersed in the central dispersion point. The parameters of sonication were as follows: volume of 50 mL, power-limited (50 W), automatically controlled frequency, time (6 times 5 min with intermittent breaks for cooling down to 15 °C to reduce the loss of the substance and possible thermal reduction of GO). After the last cooling, the samples were combined with the BSA solution (622.17 mg L–1). Earlier, BSA was dissolved in water (3.733 g L^{-1}). The BSA colloidal solution was stirred for 15 min, filtered with a filter paper, and then diluted by six times in ultrapure water. Such a solution (90 mL) was combined with the GO dispersion (50 mL) and mixed for 15 min (carefully, to avoid foaming). Subsequently, BSA-GO dispersions were combined with the modified KH solution (1 volumetric unit of BSA-GO dispersion with 4 volumetric units of the KH solution) and mixed for 15 min on a magnetic stirrer. KH reagents were diluted in ultrapure water whose volume was reduced by 20% to receive final electrolyte concentrations later, after combining with BSA-GO dispersions. The final concentrations were as follows: 10 µg mL^{-1} + 80 mg mL^{-1} BSA (all GO types), and 30 µg mL^{-1} + 240 mg mL^{-1} BSA (only GO2 and GO3). At a high concentration of GO, the power of sonication was increased to 100 W (6x5 min, volume of 50 mL). The modified KH buffer contained NaCl (124 mmol L^{-1}), KCl (4.2 mmol L_{-1}), $NaHCO_3$ (15 mmol L^{-1}), $MgSO_4$ anhydrous (1.5 mmol L^{-1}), KH_2PO_4 (1.2 mmol L^{-1}), $CaCl_2$ anhydrous (1.5 mmol L–1), sodium pyruvate (5.0 mmol L^{-1}), and anhydrous glucose (5.6 mmol L^{-1}). Finally, the KH buffer with BSA and GO was divided into two parts: the main part (usually 1500 mL) was left and stirred, whereas 500 mL was mixed (5 min) with the previously prepared indomethacin stock solution (to the final concentration of 5 µM). Next, 500 mL was divided into 200 mL and 300 mL. A microvolume of bradykinin stock solution was added to 200 mL of the solution (the final bradykinin concentration was 100 nM, while indomethacin 5 µM).

All stock dispersions were declared by producers as stable, with the following particle mean size distributions that we determined: **GO1: Z-Ave 3557 nm with polydispersity index (PDI) 0.916; GO2: Z-Ave 4363 nm with PDI 0.668; GO3: Z-Ave: 1077 nm with PDI 0.717, and GO4: Z-Ave 3386 nm with PDI 0.844** (averaged Zetasizer data from multiple measurements). The particle sizes in stock aqueous dispersions were as large as to

increase the probability of aggregation and coronary vessel blockage due to the microvessel size of approximately 5,000 nm.[1] During preparation, a reduction in polydispersity (PDI) and the particle size was observed in all tested dispersions (Table S1). To assess closely the particle diameters, two methods of particle size calculation were used: Z-ave to express the particle size in a single digit and particle-size distribution of the main particle populations. In our study, the homogeneity of the particle size of GO-BSA-KH dispersions was high. Only in two (i.e., G1-10, G3-30) out of the ten experimental groups, the percentage of the most frequent particle fraction was below 90%, whereas in the six analyzed groups, it was higher than 99%. The high homogeneity was crucial for analyzing the influence of GO particle size on the cardiac function.

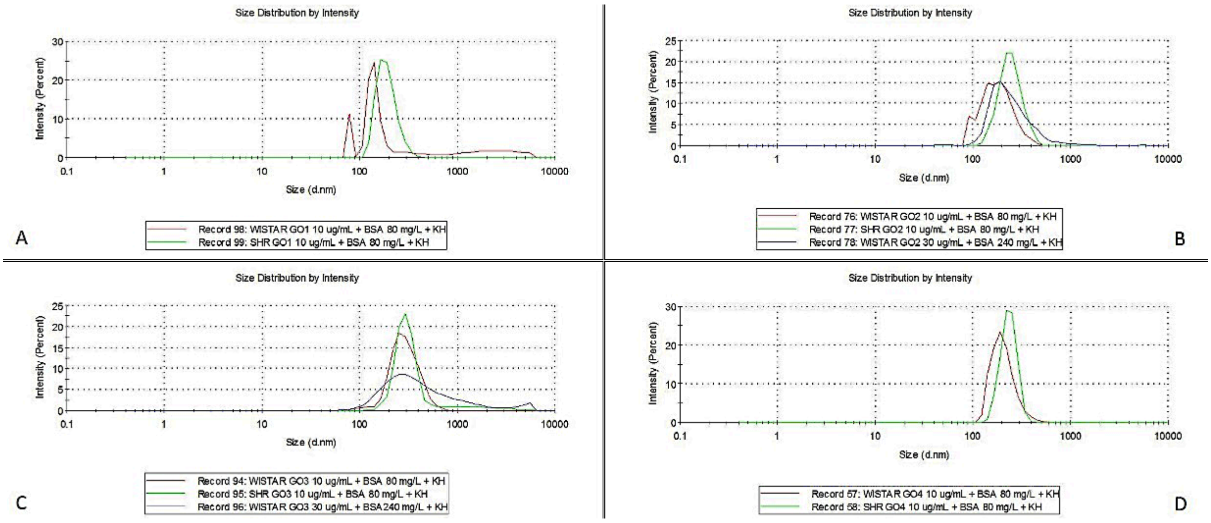

Figure S1. The averaged DLS particle size measurement results of the particle populations used in heart perfusion in evaluated groups of experiments. A: GO1 in SHR and control animals; B: GO2 in SHR rats and in Wistar animals (10 μg mL^{-1} red, 30 μg mL^{-1} blue); C: GO3 in SHR and control animals (10 μg mL^{-1} red, 30 μg mL^{-1} blue); D: GO4 in SHR and control animals

Table S1. GO nanoparticle size and distribution analysis in ready-to-use dispersions measured with DLS in studied groups. A: The comparison of particle diameters observed in dispersions used in SHR experiments (10 µg mL^{-1} GO), B: Particle diameters observed in Wistar experiments (10 µg mL^{-1} GO); C: The comparison of particle diameters observed in all experiments with GO2; D: The comparison of particle diameters observed in all experiments with GO3.

A

**Study group**	**G1-10SHR**	**G2-10SHR**	**G3-10SHR**	**G4-10SHR**	$p^{\times}$
n	18	21	21	21	
Z-Ave (nm)	419.6 ± 135.9^{1}	398.2 ± 70.1^{4,2}	463.3 ± 117.4^{4,3}	546.9 ± 116.0^{1,2,3}	<0.001
PDI	0.38 ± 0.08^{1,2}	0.37 ± 0.06 ^{3,4}	0.41 ± 0.05^{1,3,5}	0.46 ± 0.07^{2,4,5}	<0.001
Particle size of the main particle population (nm)	191.3 ± 30.8^{1,3,4}	248.1 ± 43.8^{2,4}	431.8 ± 346.5^{1,2,5}	235.2 ± 24.5^{3,5}	<0.001
Main particle population (%)	100±0	100±0	99.2 ± 2.8	100±0	0.11

B

**Study group**	**G1-10**	**G2-10**	**G3-10**	**G4-10**	$p^{\times}$
n	18	30	27	18	
Z-Ave (nm)	710.5 ± 330.7^{1,3,4}	601.03 ± 365.8^{2,4}	409.5 ± 59.9^{1,2}	477.8 ± 184.9^{3}	0.002
PDI	0.62 ± 0.19^{1,2,3}	0.48 ± 0.19^{3}	0.43 ± 0.06^{1}	0.43 ± 0.12^{2}	0.011
Particle size of the main particle population (nm)	652.1 ± 992.4^{1}	181.4 ± 60.7^{2}	305.7 ± 47.1^{1,2,3}	208.0 ± 43.6^{3}	<0.001
Main particle population (%)	86.74 ± 20.3^{1,2,3}	99.9 ± 0.26^{3}	98.4 ± 4.15^{1}	99.4 ± 2.29^{2}	0.03

C

**Study group**	**G2-10**	**G2-10SHR**	**G2-30**	**x**	$p^{\times}$
n	18	21	24	x	
Z-Ave (nm)	601.03 ± 365.8^{1}	398.2 ± 70.1^{1}	452.05 ± 134.3	x	0.049
PDI	0.48 ± 0.19	0.37 ± 0.06	0.42 ± 0.07	x	0.06
Particle size of the main particle population (nm)	181.4 ± 60.7^{1,2}	248.1 ± 43.8^{2}	271.9 ± 138.4^{1}	x	<0.001
Main particle population (%)	99.95 ± 0.26^{1}	100±0^{2}	92.46 ± 14.82^{1,2}	x	0.001

D

**Study group**	**G3-10**	**G3-10SHR**	**G3-30**	**x**	$p^{\times}$
n	27	21	24	x	
Z-Ave (nm)	409.5 ± 59.9	463.3 ± 117.4^{1}	363.2 ± 89.2^{1}	x	0.011
PDI	0.43 ± 0.06	0.41 ± 0.05	0.41 ± 0.05	x	0.56
Particle size of the main particle population (nm)	305.7 ± 47.0^{1}	431.8 ± 346.5^{2}	530.1 ± 319.5^{1,2}	x	0.031
Main particle population (%)	98.4 ± 4.15^{1}	99.2 ± 2.83^{2}	86.75 ± 15.63^{1,2}	x	<0.001

Abbreviations: DLS, dynamic light scattering; Z-Ave, average particle size in the sample; PDI, polydispersity index; Particle size of main particle population: particle size by intensity in the main population/fraction of particles (nm); Main particle population (%): area under the particle size distribution curve representing the percentage of the dominant particle fraction (100% is observed in single-size particle population); n, number of particle size measurements. $p^{\times}$ Kruskal-Wallis rank sum test; Superscripts: ^{1},^{2},^{3},^{4} represent $p < 0.05$ in Dunn test for post-hoc analysis between the values with the same sign (comparison in rows).

Bovine serum albumin and graphene oxide interaction

The reports confirmed the modification of the bovine serum albumin (BSA) activity and reduction of GO toxicity in the BSA-GO complexes.[2,3] BSA alpha-helix content decreased to 59%, 47%, and 18% upon the formation of a protein complex with GO of mean particle size 955, 475, and 285 nm, respectively.[4] Among others, BSA, was reported to be protein-firmly-binding to nanoparticles.[5] With its isoelectric point at pH 4.6, BSA is negatively charged at pH between 5 and 9.[6] Although its total particle charge at pH 7 is negative (–17)[7], it expresses positively charged lysine residues (Lys537, Lys535) with an isoelectric point[6] at pH 11 that can penetrate the water layer and create a hydrogen bond with oxygen atoms at the negatively charged surface of surrounding particles.

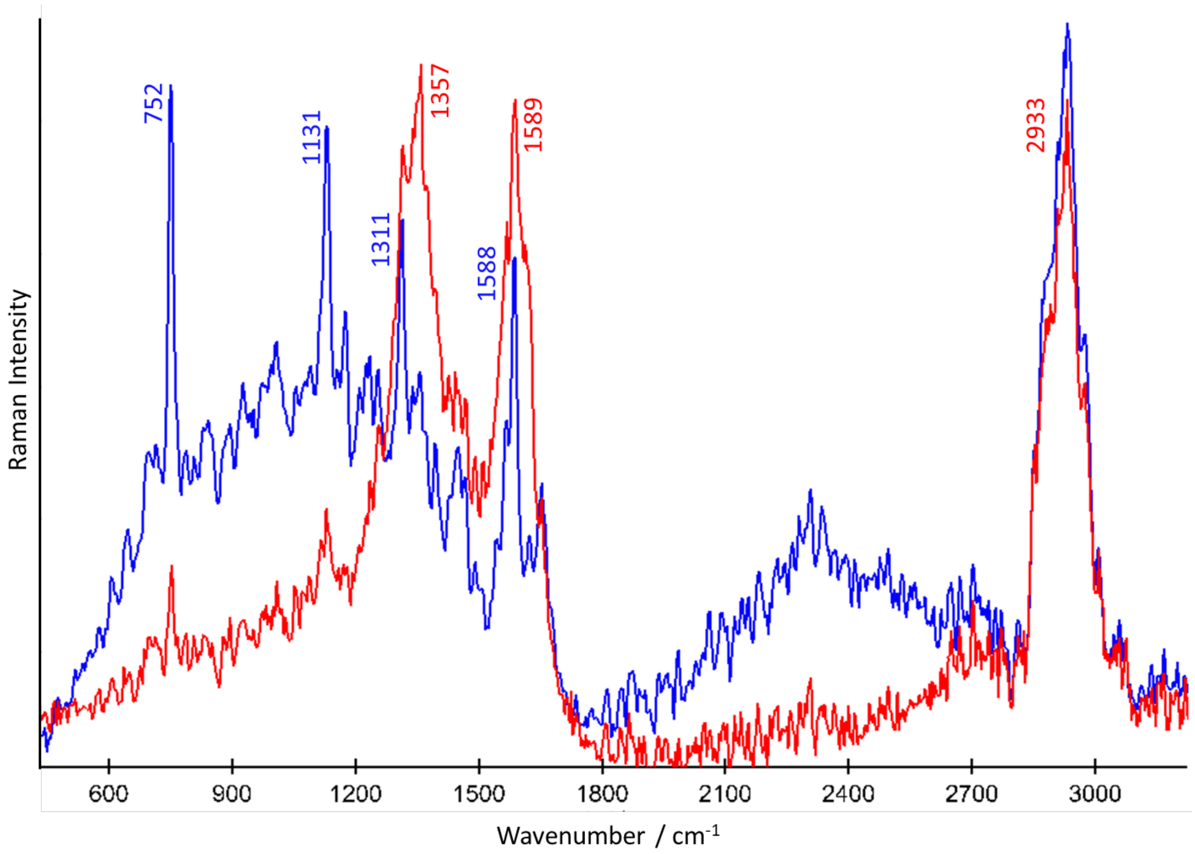

Figure S2. Representative Raman spectra of classes separated as a result of CA of the rat heart tissue containing GO. The average spectra of the GO class (red) and tissue class (blue) extracted from the GO1 sample (CA: K-means, Manhattan distance, 3 classes).

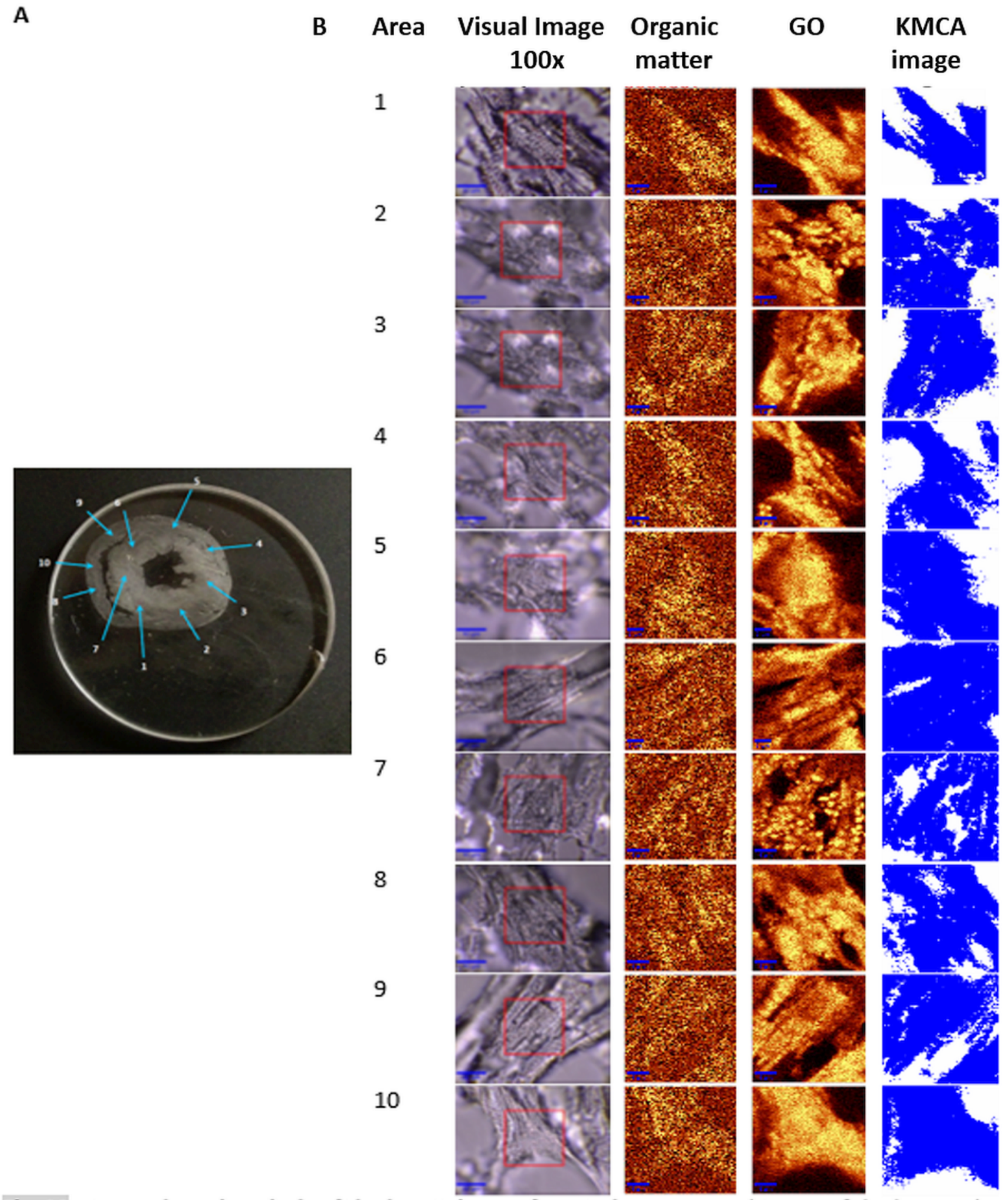

Figure S3. Raman-based analysis of the heart tissue of control rats. A visual image of the hearts showing the analyzed areas (**A**) and the analysis of the tissue fragments (**B**): representative visual images (100×), Raman distribution images of the organic matter and GO in the tissue (obtained by integration of the signals in the 3030-2830 cm^{-1} and 1657-1538 cm^{-1} ranges, respectively) and CA image showing classes assigned to GO (red), tissue (blue) and background (no signal, white).

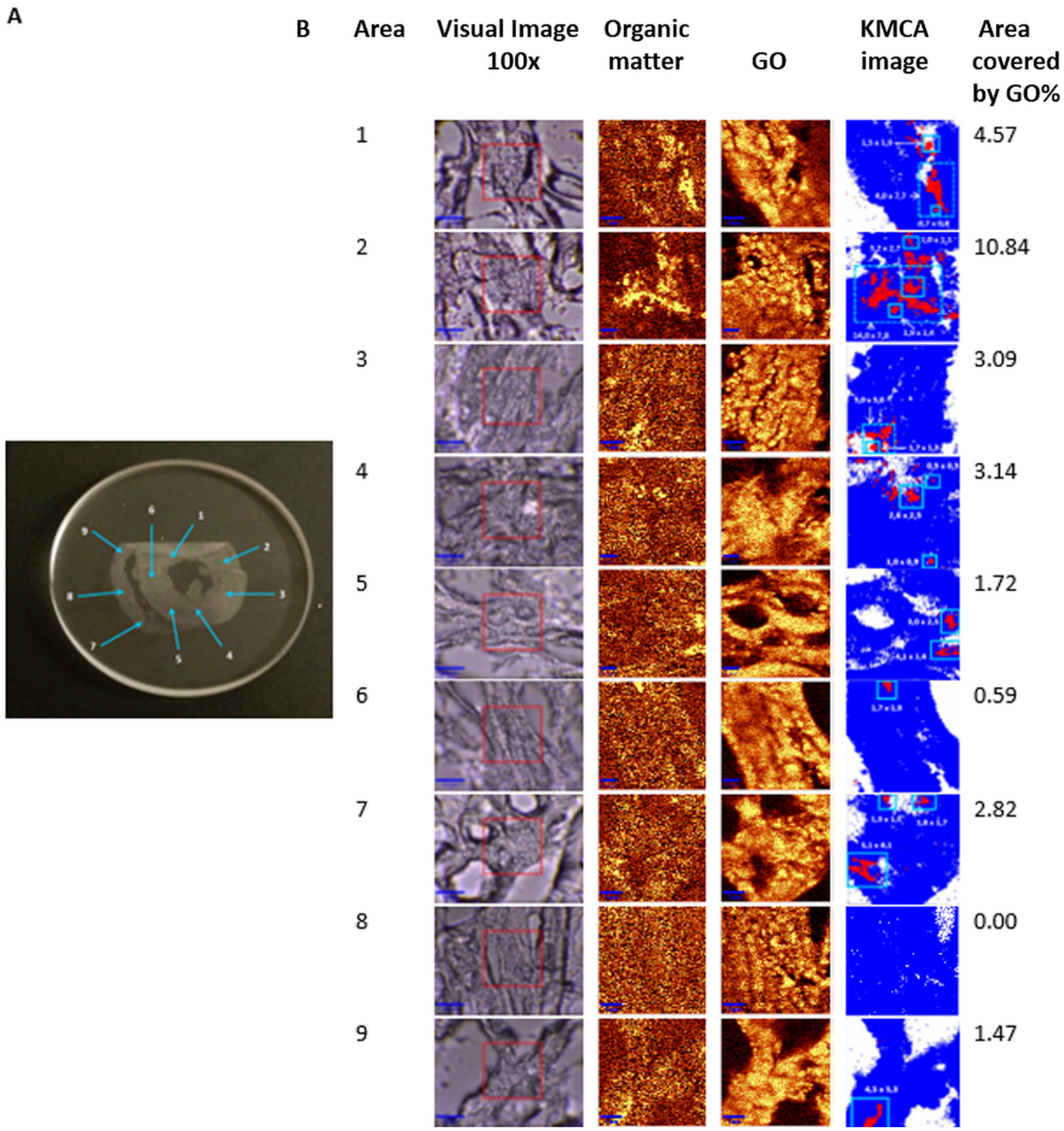

Figure S4. Raman-based analysis of a rat heart perfused with GO1 (Z-ave 700 nm). A visual image of the heart showing the analyzed areas (**A**) and analysis of the tissue fragments (**B**): representative visual images (100×), Raman distribution images of the organic matter and GO in the tissue (obtained by integration of the signals in the 3030-2830 cm^{-1} and 1657-1538 cm^{-1} ranges, respectively) and CA image showing classes assigned to GO (red), tissue (blue) and background (no signal, white).

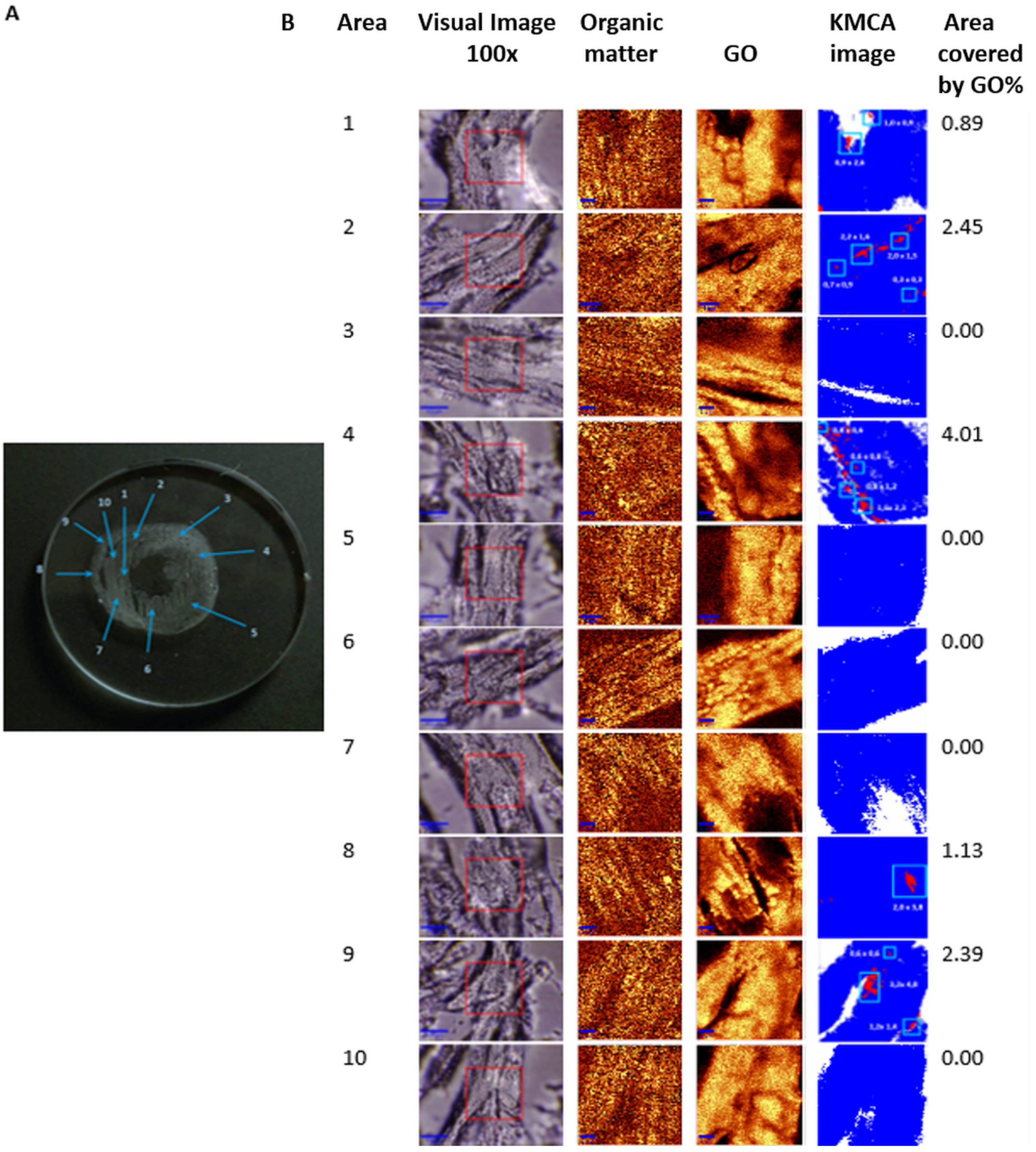

Figure S5. Raman-based analysis of a rat heart perfused with GO2 (Z-ave 364 nm). A visual image of the heart showing the analyzed areas (**A**) and analysis of the tissue fragments (**B**): representative visual images (100×), Raman distribution images of the organic matter and GO in the tissue (obtained by integration of the signals in the 3030-2830 cm^{-1} and 1657-1538 cm^{-1} ranges, respectively) and CA image showing classes assigned to GO (red), tissue (blue) and background (no signal, white).

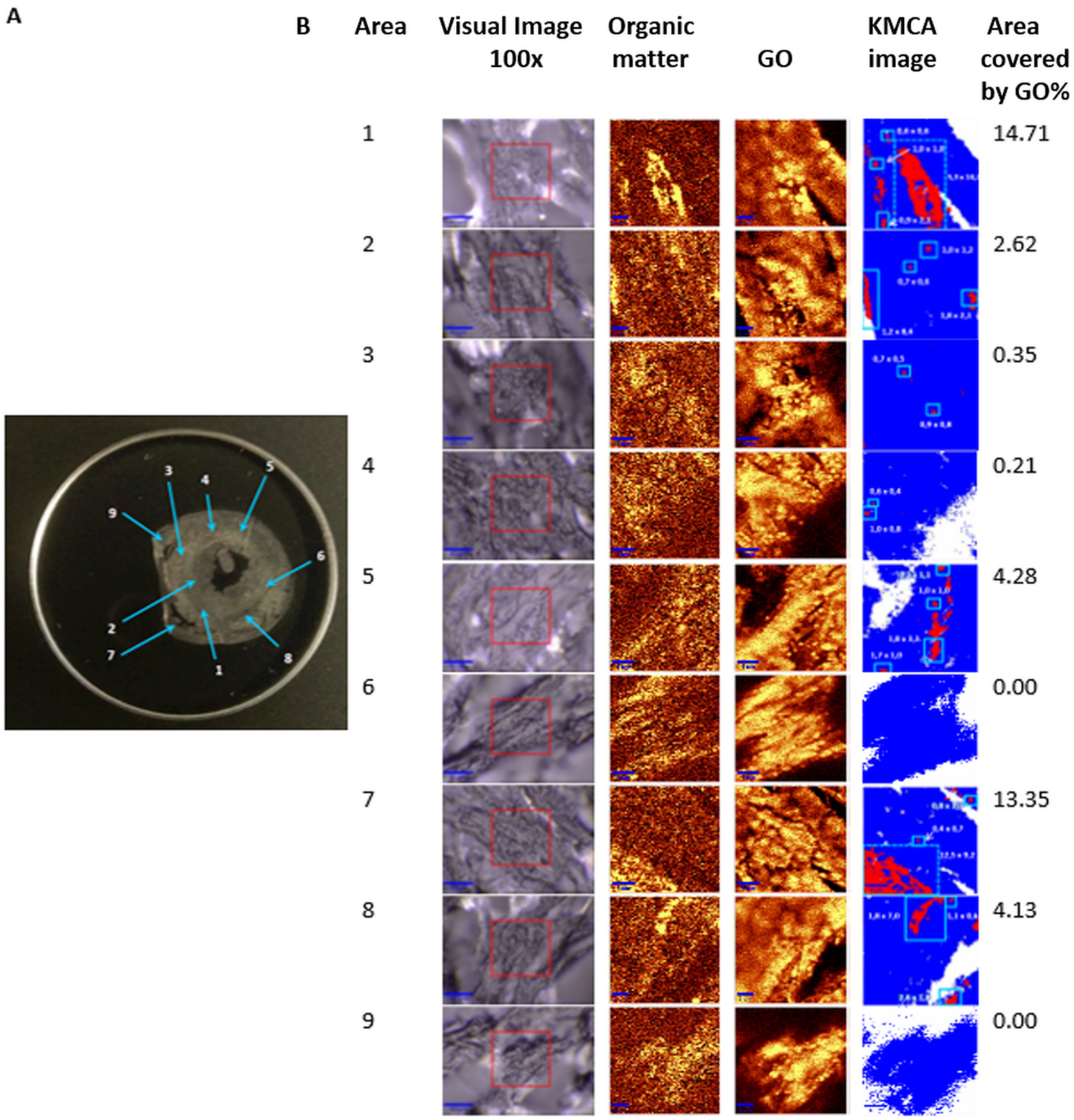

Figure S6. Raman-based analysis of a rat heart perfused with GO2 (Z-ave 473 nm). A visual image of the heart showing the analyzed areas (**A**) and analysis of the tissue fragments (**B**): representative visual images (100×), Raman distribution images of the organic matter and GO in the tissue (obtained by integration of the signals in the 3030-2830 cm^{-1} and 1657-1538 cm^{-1} ranges, respectively) and CA image showing classes assigned to GO (red), tissue (blue) and background (no signal, white).

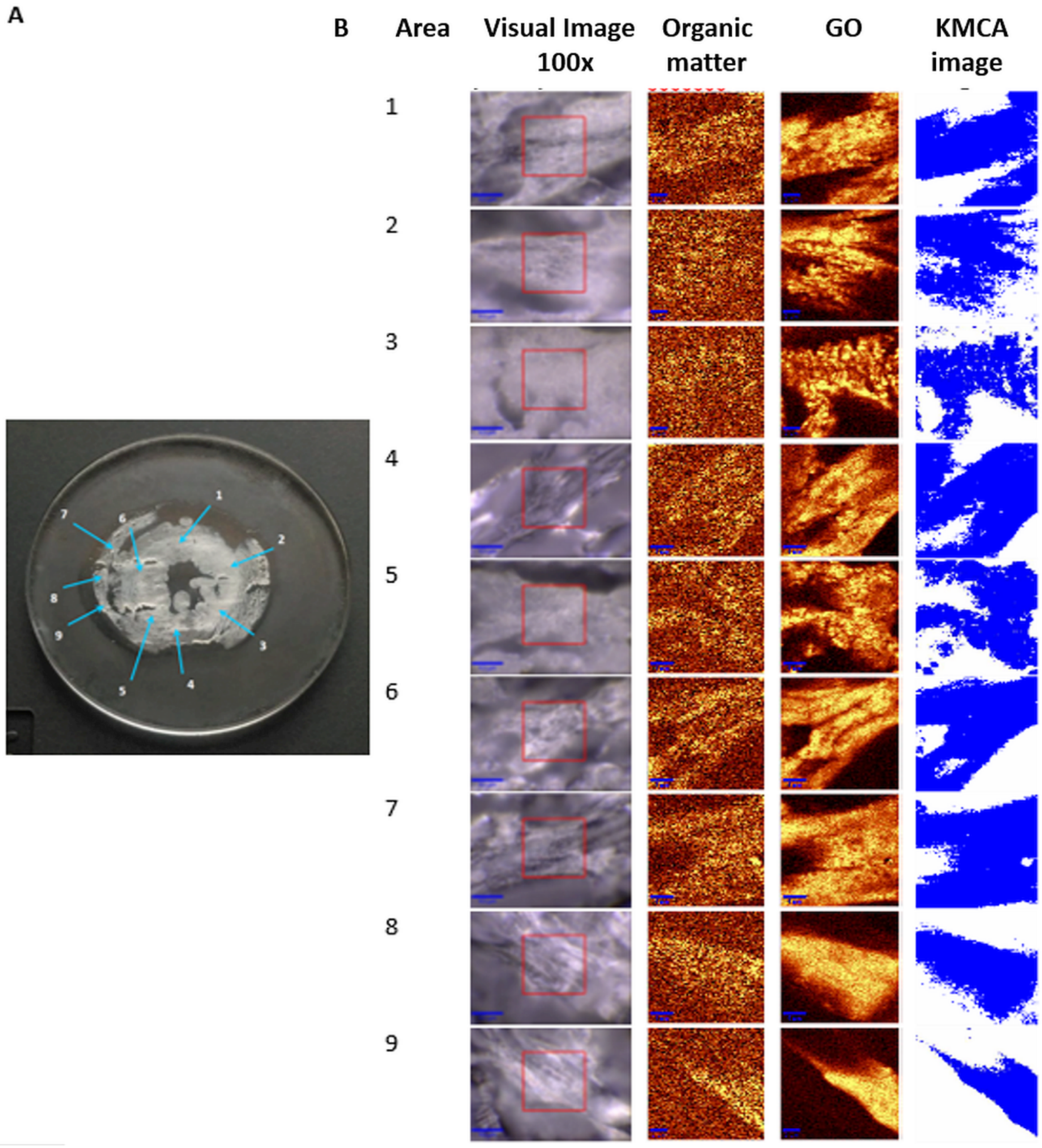

Figure S7. Raman-based analysis of a rat heart perfused with GO3 (filtered, Z-ave 420nm). A visual image of the heart showing the analyzed areas (**A**) and analysis of the tissue fragments (**B**): representative visual images (100×), Raman distribution images of the organic matter and GO in the tissue (obtained by integration of the signals in the 3030-2830 cm^{-1} and 1657-1538 cm^{-1} ranges, respectively) and CA image showing classes assigned to GO (red), tissue (blue) and background no signal, white).

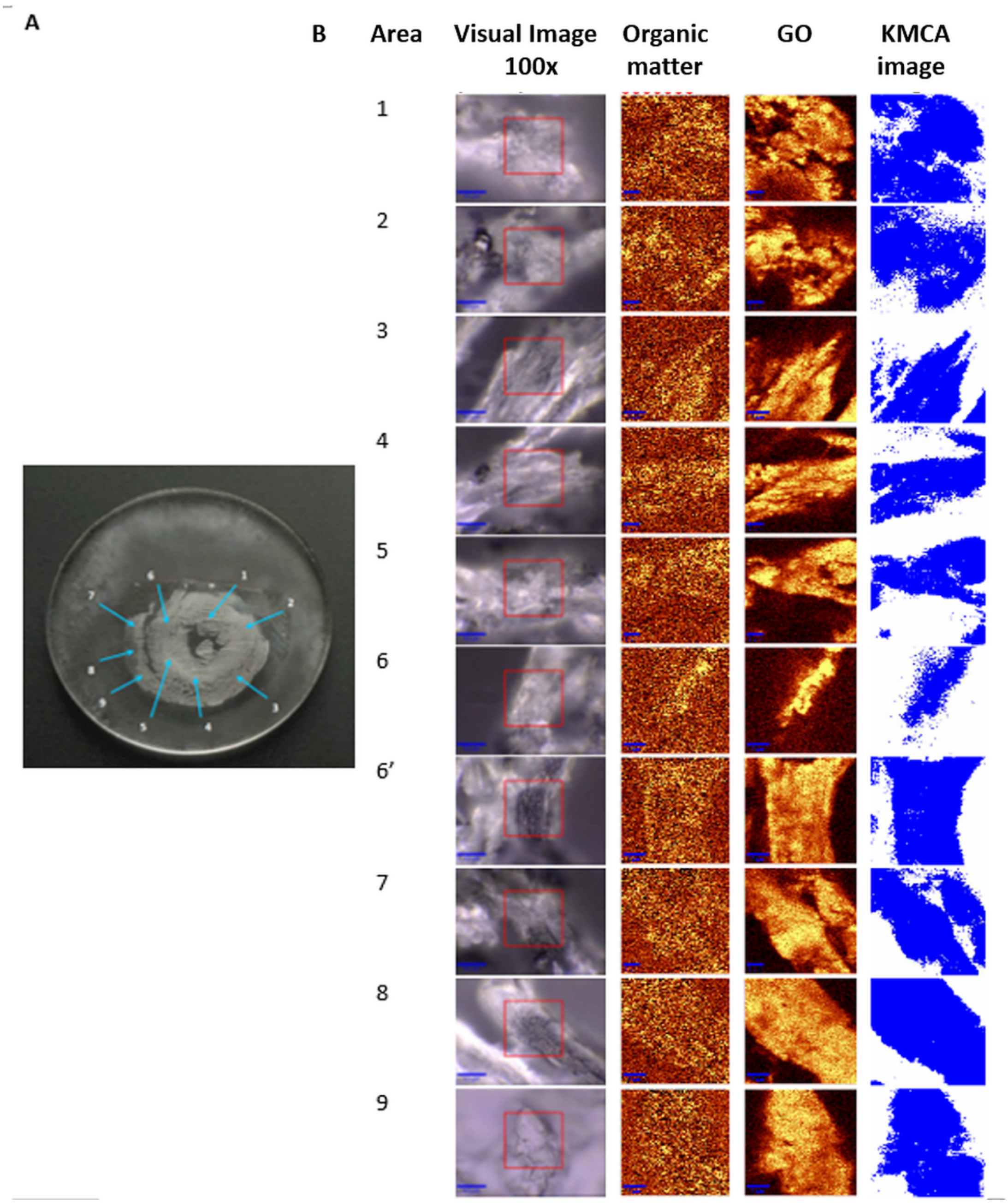

Figure S8. Raman-based analysis of a rat heart perfused with GO3 (non-filtered, Z-ave 420 nm). A visual image of the heart showing the analyzed areas (**A**) and analysis of the tissue fragments (**B**): representative visual images (100×), Raman distribution images of the organic matter and GO in the tissue (obtained by integration of the signals in the 3030-2830 cm^{-1} and 1657-1538 cm^{-1} ranges, respectively) and CA image showing classes assigned to GO (red), tissue (blue) and background (no signal, white).

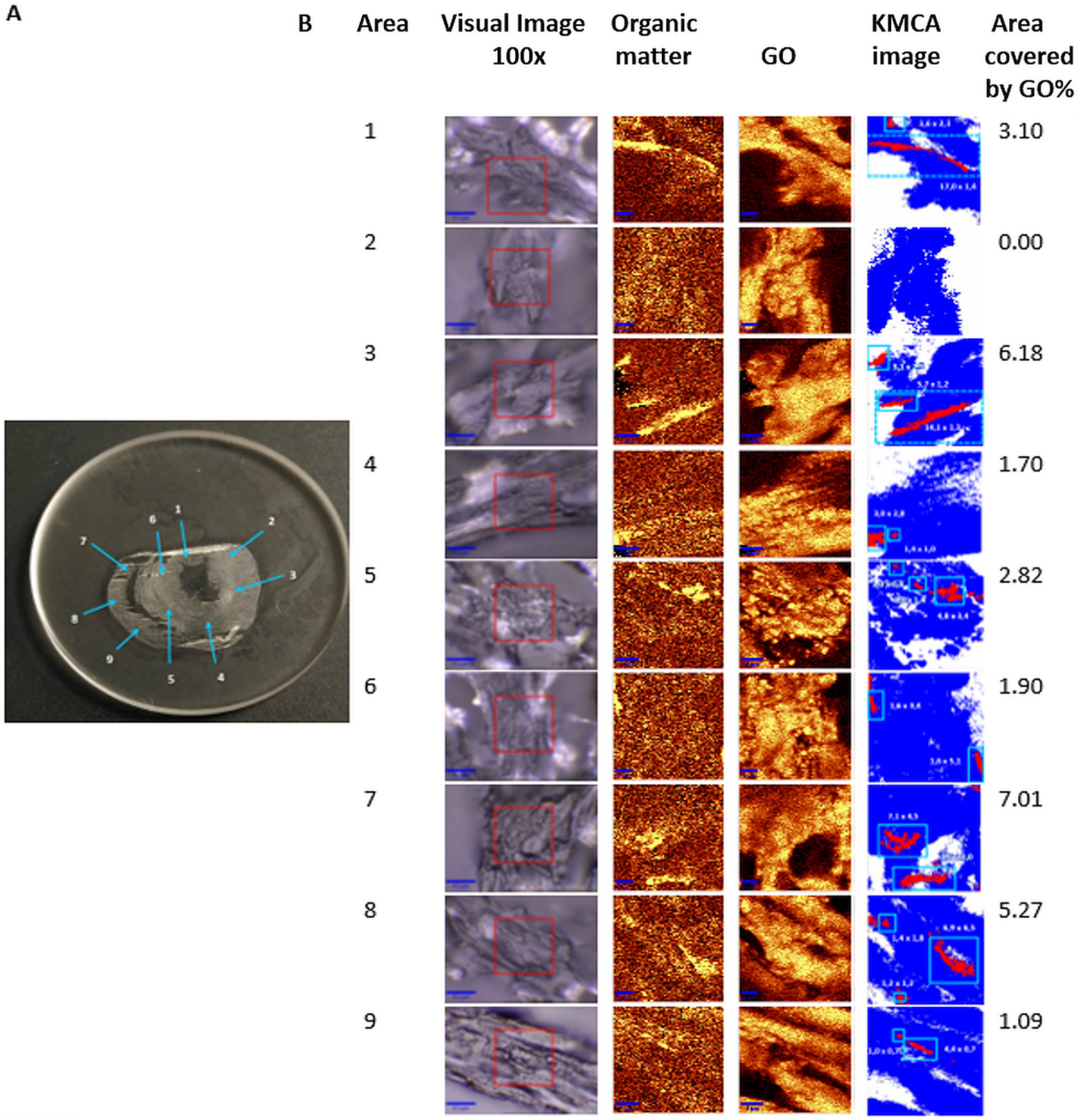

Figure S9. Raman-based analysis of a rat heart perfused with GO4. A visual image of the heart showing the analyzed areas (**A**) and analysis of the tissue fragments (**B**): representative visual images (100×), Raman distribution images of the organic matter and GO in the tissue (obtained by integration of the signals in the 3030-2830 cm^{-1} and 1657-1538 cm^{-1} ranges, respectively) and CA image showing classes assigned to GO (red), tissue (blue) and background (no signal, white).

Table S2. Echocardiographic diameters of the Wistar and SHR hearts measured before experiments at the parasternal long axis view in B-Mode. Abbreviations: B-mode, brightness mode, standard 2D grayscale view; IVSd, IVSs Interventricular septum diameters: diastolic and systolic; LVIDd, LVIDs, left ventricular internal diameters: diastolic and systolic; LVPWd, LVPWs, left ventricular posterior wall diameters: diastolic and systolic; na, not analyzed. $^{3}p<0.05$ for the marked parameters, ^{1,2,4,5,6} $p<0.01$ for the marked parameters

**Echo view and mode**			**Parasternal long axis view, B-mode**						
**Echo parameters**			IVSd	IVSs	LVIDd	LVIDs	LVPWd	LVPWs	body weight (b.w.)
**all**	Wistar	mean	2.001	3.515	14.672	12.346	2.163	3.211	na
**all**	SHR	mean	2.344	3.765	14.301	12.176	2.142	3.688	na
**highest IVSd**									
**n=15**	Wistar	mean	2.35[1]	3.951	14.942[2]	12.025	2.461[3]	3.491	na
		SD	0.126	0.380	0.517	1.406	0.370	0.552	
**n=15**	SHR	mean	2.619[1]	3.833	14.151[2]	12.100	2.113[3]	3.733	na
		SD	0.196	0.284	0.677	1.057	0.326	0.363	
**highest IVSs**									
**n=15**	Wistar	mean	2.161[4]	4.212	14.85[5]	11.819	2.412	3.498	na
		SD	0.231	0.208	0.644	1.394	0.376	0.532	
**n=15**	SHR	mean	2.451[4]	4.062	14.107[5]	11.782	2.163	3.786	na
		SD	0.332	0.170	0.644	1.199	0.301	0.388	
**highest body weight**									
**n=15**	Wistar	mean	2.180	3.692	14.973	12.029	2.416	3.615	477.07[6]
		SD	0.262	0.479	0.475	1.211	0.388	0.393	27.580
**n=15**	SHR	mean	2.360	3.760	14.774	12.758	2.266	3.718	323.053[6]
		SD	0.221	0.295	0.390	0.977	0.315	0.409	6.585

Table S3. The effects of GO2 in three groups: G2-10, G2-10SHR, and G2-30.

PARAMETER	GROUP	CONTROL	GO perfusion start	GO perfusion half-time	GO perfusion end
	Exp. stage	cwh1-2	twh2-0	twh2-1	twh2-2
CFM coronary flow mean (mL min^{-1})	G2-10	19 ± 7	17 ± 3	14 ± 4	11 ± 5[1]
	G2-10SHR	14 ± 4	12 ± 4*	14 ± 3	12 ± 5*
	G2-30	15 ± 4	15 ± 5	11 ± 3*	12 ± 2
AoFM aortic flow mean (mL min^{-1})	G2-10	39 ± 13	39 ± 16	40 ± 17	39 ± 14
	G2-10SHR	31 ± 10	32 ± 11	31 ± 9	32 ± 12
	G2-30	37 ± 14	34 ± 8	29 ± 8	28 ± 4
HRAP heart rate from aortic pressure (1 min^{-1})	G2-10	231 ± 49 [2]	241 ± 14	232 ± 41	213 ± 50
	G2-10SHR	157 ± 42 [2]	156 ± 51	177 ± 65	158 ± 53
	G2-30	227 ± 44	229 ± 35	217 ± 29	202 ± 31
APM aortic pressure mean (mmHg)	G2-10	91 ± 8	91 ± 9	90 ± 9	87 ± 8*
	G2-10SHR	84 ± 5	86 ± 7	86 ± 6*	83 ± 6
	G2-30	87 ± 8	90 ± 4	85 ± 3	85 ± 4[1]

Abbreviations: exp stage, experimental stage. *p<0.05 with cwh1-2, [1,2] p< 0.05 with cwh2-0.

Table S4. The effects of GO3 in three groups: **G3-10, G3-10SHR and G3-30**.

PARAMETER	GROUP	CONTROL	GO perfusion start	GO perfusion half-time	GO perfusion end
	Exp. stage	cwh1-2	twh2-0	twh2-1	twh2-2
CFM coronary flow mean ($mL\ min^{-1}$)	G3-10	19 ± 5 [2]	19 ± 7	15 ± 8	15 ± 6
	G3-10SHR	13 ± 4 [2,3]	12 ± 4	10 ± 5*	10 ± 4*[,1]
	G3-30	17 ± 5 [3]	16 ± 4	15 ± 4	15 ± 5
AoFM aortic flow mean ($mL\ min^{-1}$)	G3-10	42 ± 12	39 ± 12	43 ± 17	31 ± 22
	G3-10SHR	33 ± 8	33 ± 7	27 ± 6*	25 ± 8*[,1]
	G3-30	37 ± 10	31 ± 8*	33 ± 9	30 ± 3
HRAP heart rate from aortic pressure ($1\ min^{-1}$)	G3-10	230 ± 37	234 ± 11	233 ± 11	232 ± 25
	G3-10SHR	208 ± 39	212 ± 38	199 ± 35	188 ± 26[1,4]
	G3-30	226 ± 36	230 ± 31	223 ± 33	228 ± 48[4]
APM aortic pressure mean (mmHg)	G3-10	91 ± 6	92 ± 4	90 ± 3	85 ± 7
	G3-10SHR	86 ± 4	86 ± 4	83 ± 3	83 ± 5*[,1]
	G3-30	92 ± 5	91 ± 3	89 ± 3	89 ± 4

Abbreviations: exp. stage, experimental stage. *$p<0.05$ with control cwh1-2, [1] $p< 0.05$ with cwh2-0. [2,3,4] $p<0.05$ with the same superscript.

Routes of GO administration and toxicology of the GO.

GO circulatory effects are an important part of our study. The oral administration route was tested on mice with 3-6 µm GO flakes dispersed in water. The doses used ranged between 30 to 60 and 120 mg kg^{-1}. No obvious negative effects were observed in the hearts, livers, and kidneys of the treated animals, whereas the mice administered with the highest dose of GO demonstrated lower body weight, a shorter gut, and a tendency to apoptosis in the intestine wall. Moreover, the microbiota of the gut of the treated animals was modified.[8] Inhalatory, single nose-only administration of GO (particle size between 150 and 250 nm, with 20 wt.% oxygen and 57 wt.% carbon) caused at the dose of 0,087 mg kg^{-1} b.w. only a significant increase of MMP-9 (day 1), IL-18, and TGF-ß1 (day 7), but no damage to the testes, kidneys, spleen, liver, lungs, and brain. These changes returned to normal on the 7th post-exposure day. The temporary elevation of these markers could be related to macrophage activation and GO phagocytosis.[9] In another study,[10] the repeated nose-only inhalations of GO in concentrations of 0.76, 2.60, and 9.78 mg m^{-3} caused concentration of macrophages in proximity of ingested GO (still present on the 21st day of observation), no changes in lung parenchyma, liver, and kidneys, and no blood cell count modification; also, a decrease in cholesterol, creatinine, lactate dehydrogenase, albumin, sodium, and magnesium blood levels were found. In this study, the inhaled aerosol contained GO nanoparticles of stacked

platelet structure with particle sizes ranging from 5.4 to 209.1 nm and 35-40 wt.% of oxygen. The mechanisms of lung injury after intravenous GO administration were studied by Zhang *et al.*[11] In this study, it was shown that low doses of GO (5 and 10 mg kg^{-1}) did not cause significant lung injury, whereas higher doses (50 and 100 mg kg^{-1}) provoked lung edema, tissue changes, and an inflammatory response with increased levels of oxidative products and inflammatory mediators. The inhibition of autophagy with chloroquine (CLQ) and 3-methyladenine (3-MA) reduced both lung injury and oxidative stress. In other studies, GO was cytotoxic to human fibroblasts and lung epithelial cells at concentrations above 20 μg mL^{-1} whereas to A549 cells at a concentration of 50 μg mL^{-1}.[12,13]

REFERENCES

1. Barrs RW, Jia J, Silver SE, Yost M, Mei Y. Biomaterials for Bioprinting Microvasculature. *Chem Rev.* 2020;120(19):10887-10949. doi:10.1021/acs.chemrev.0c00027

2. Zhang H, Zhu Z, Wang Y, Fei Z, Cao J. Changing the activities and structures of bovine serum albumin bound to graphene oxide. *Appl Surf Sci.* 2018;427:1019-1029. doi:10.1016/j.apsusc.2017.08.130

3. Björkman Å. Thermische Klärschlammbehandlung. *Schweizerische Zeitschrift für Hydrol.* 1969;31(2):632-645. doi:10.1007/BF02543692

4. Šimšíková M. Interaction of graphene oxide with albumins: Effect of size, pH, and temperature. *Arch Biochem Biophys.* 2016;593:69-79. doi:10.1016/j.abb.2016.02.015

5. Neagu M, Piperigkou Z, Karamanou K, et al. Protein bio-corona: critical issue in immune nanotoxicology. *Arch Toxicol.* 2017;91(3):1031-1048. doi:10.1007/s00204-016-1797-5

6. Sun B, Zhang Y, Chen W, Wang K, Zhu L. Concentration Dependent Effects of Bovine Serum Albumin on Graphene Oxide Colloidal Stability in Aquatic Environment. *Environ Sci Technol.* 2018;52(13):7212-7219. doi:10.1021/acs.est.7b06218

7. Kubiak-Ossowska K, Jachimska B, Mulheran PA. How Negatively Charged Proteins Adsorb to Negatively Charged Surfaces: A Molecular Dynamics Study of BSA Adsorption on Silica. *J Phys Chem B.* 2016;120(40):10463-10468. doi:10.1021/acs.jpcb.6b07646

8. Shen J, Dong J, Zhao J, et al. The effects of the oral administration of graphene oxide on the gut microbiota and ultrastructure of the colon of mice. *Ann Transl Med.* 2022;10(6):278-278.

doi:10.21037/atm-22-922

9. Han SG, Kim JK, Shin JH, et al. Pulmonary responses of sprague-dawley rats in single inhalation exposure to graphene oxide nanomaterials. *Biomed Res Int*. 2015;2015:1-9. doi:10.1155/2015/376756

10. Kim YH, Jo MS, Kim JK, et al. Short-term inhalation study of graphene oxide nanoplates. *Nanotoxicology*. 2018;12(3):224-238. doi:10.1080/17435390.2018.1431318

11. Zhang L, Ouyang S, Zhang H, et al. Graphene oxide induces dose-dependent lung injury in rats by regulating autophagy. *Exp Ther Med*. 2021;21(5):462. doi:10.3892/etm.2021.9893

12. De Marzi L, Ottaviano L, Perrozzi F, et al. Flake size-dependent cyto and genotoxic evaluation of graphene oxide on in vitro A549, CaCo2 and vero cell lines. *J Biol Regul Homeost Agents*. 2014;28(2):281-289.

13. Chang Y, Yang S-T, Liu J-H, et al. In vitro toxicity evaluation of graphene oxide on A549 cells. *Toxicol Lett*. 2011;200(3):201-210. doi:10.1016/j.toxlet.2010.11.016
